# Scavenger-Supported Photocatalytic Evidence of an Extended Type I Electronic Structure of the TiO_2_@Fe_2_O_3_ Interface

**DOI:** 10.1021/acsami.2c06404

**Published:** 2022-08-15

**Authors:** Anita Trenczek-Zajac, Milena Synowiec, Katarzyna Zakrzewska, Karolina Zazakowny, Kazimierz Kowalski, Andrzej Dziedzic, Marta Radecka

**Affiliations:** † Faculty of Materials Science and Ceramics, AGH University of Science and Technology, Krakow 30-059, Poland; ‡ Faculty of Computer Science, Electronics and Telecommunications, AGH University of Science and Technology, Krakow 30-059, Poland; § Faculty of Metals Engineering and Industrial Computer Science, AGH University of Science and Technology, Krakow 30-059, Poland; ∥ Institute of Physics, College of Natural Sciences, University of Rzeszow, Rzeszow 35-310, Poland

**Keywords:** TiO_2_, Fe_2_O_3_, heterostructures, band diagram, interface, electron transfer, photocatalysis

## Abstract

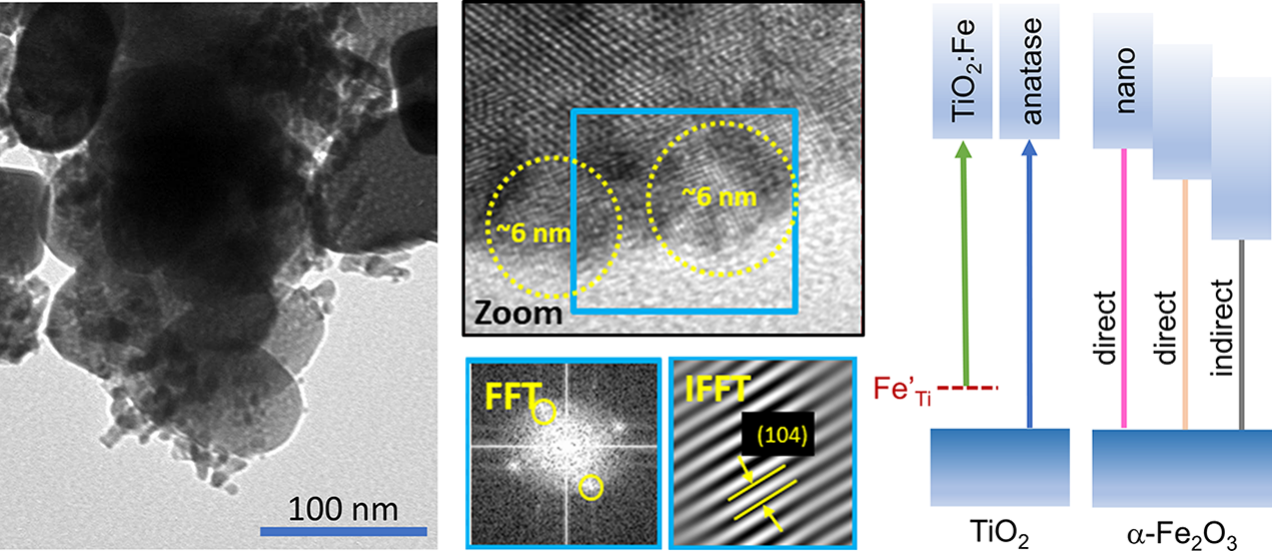

Heterostructures of TiO_2_@Fe_2_O_3_ with a specific electronic structure and morphology enable us to control the interfacial charge transport necessary for their efficient photocatalytic performance. In spite of the extensive research, there still remains a profound ambiguity as far as the band alignment at the interface of TiO_2_@Fe_2_O_3_ is concerned. In this work, the extended type I heterojunction between anatase TiO_2_ nanocrystals and α-Fe_2_O_3_ hematite nanograins is proposed. Experimental evidence supporting this conclusion is based on direct measurements such as optical spectroscopy, X-ray photoemission spectroscopy, scanning electron microscopy, high-resolution transmission electron microscopy (HRTEM), and the results of indirect studies of photocatalytic decomposition of rhodamine B (RhB) with selected scavengers of various active species of OH^•^, h^•^, e^–^, and ^•^O_2_^–^. The presence of small 6–8 nm Fe_2_O_3_ crystallites at the surface of TiO_2_ has been confirmed in HRTEM images. Irregular 15–50 nm needle-like hematite grains could be observed in scanning electron micrographs. Substitutional incorporation of Fe^3+^ ions into the TiO_2_ crystal lattice is predicted by a 0.16% decrease in lattice parameter a and a 0.08% change of c, as well as by a shift of the Raman E_g(1)_ peak from 143 cm^–1^ in pure TiO_2_ to 149 cm^–1^ in Fe_2_O_3_-modified TiO_2_. Analysis of O 1s XPS spectra corroborates this conclusion, indicating the formation of oxygen vacancies at the surface of titanium(IV) oxide. The presence of the Fe^3+^ impurity level in the forbidden band gap of TiO_2_ is revealed by the 2.80 eV optical transition. The size effect is responsible for the absorption feature appearing at 2.48 eV. Increased photocatalytic activity within the visible range suggests that the electron transfer involves high energy levels of Fe_2_O_3_. Well-programed experiments with scavengers allow us to eliminate the less probable mechanisms of RhB photodecomposition and propose a band diagram of the TiO_2_@Fe_2_O_3_ heterojunction.

## 1 Introduction

Although anatase TiO_2_ and hematite Fe_2_O_3_ have been studied for many years, completely new effects arise when the combination of both oxides is used in catalysis,^1^ photocatalysis,^2−6^ Li-ion batteries,^7,8^ gas sensors,^9,10^ and photoelectrochemical water splitting to generate green hydrogen.^11−13^ When treated separately, each of the metal oxides mentioned above offers many attractive features but suffers from fundamental drawbacks as well.

Titanium dioxide is one of the semiconductors that are the most frequently encountered in photocatalysis,^14^ solar cells,^15^ self-cleaning coatings,^16^ and gas sensors^17^ due to its non-toxicity, chemical stability, abundance, and low cost. Nevertheless, its basic disadvantage is a wide band gap E_g_ of above 3.0 eV, resulting in high transparency to the visible range of the light spectrum. Numerous attempts have been made to engineer the TiO_2_ band gap with the aim of reducing the separation between the edges of the valence and conduction bands or creating additional states in the forbidden band gap. However, the problem of better adaptation of the optical absorption of TiO_2_ to the spectrum of the Sun has never found a satisfactory solution. All efforts, including doping, largely failed due to the development of undesirable recombination centers, inherent to this method of band gap modification.

In contrast to TiO_2_, hematite Fe_2_O_3_ is a good representative of narrow-band-gap semiconductors (2.2 eV). Its absorption spectrum allows for efficient light harvesting within the visible range. Similarly to TiO_2_, it is inexpensive and environmentally friendly.^18,19^ However, fast recombination of charge carriers resulting from extremely short lifetimes of electron–hole pairs (<10 ps) and small diffusion lengths of holes (2–4 nm) inevitably contributes to the degradation of photocatalytic performance and the low efficiency of energy conversion processes. The low mobility of minority charge carriers and their limited diffusion length are considered responsible for the high surface and bulk recombination rates of charge carriers.^20^ Therefore, the biggest challenge is to restrict the recombination of the photoexcited electron and holes in order to extend their lifetime to drive much slower photocatalytic processes at the surfaces and interfaces. One of the most efficient solutions to this problem is the creation of solid-state junctions.^21^

Metal oxide heterojunctions can be categorized into type I, II, and III depending on how the band edges of two semiconductors relate to one another.^22^ Moreover, different charge carrier transfer routes have been proposed, among which Z and S schemes are the most popular.^22,23^

To take advantage of the best features of both oxides, TiO_2_@Fe_2_O_3_ heterostructures have been studied as an alternative to improve the photocatalytic performance due to charge transfer phenomena across the interfaces.^8,24−26^ Control over interfacial electronic transport is widely accepted as necessary to provide efficient operation of devices based on materials that contain numerous heterojunctions. However, to ensure the best photocatalytic decomposition of organic compounds, the type and electronic structure of the heterojunctions must be controlled as well as their morphology.

In fact, in the case of TiO_2_@Fe_2_O_3_, there remains a profound ambiguity as far as the electronic structure and its type is concerned.^27−29^ One can find different models of the configuration of electronic bands that consequently predict various mechanisms of electron and hole separation.^21^ Research results in favor of the type I^27,30−33^ and type II^19,28,34−36^ or that of the extended type I have been published.^4,29,37,38^

Formation of a type I heterojunction, where the conduction band (CB) edge of TiO_2_ is above the CB of Fe_2_O_3_ and the valence band (VB) edge of TiO_2_ is below that of Fe_2_O_3_, has been proposed.^27,30−32^ However, in this case, the photoelectrons and photoholes generated in TiO_2_ upon UV radiation would transfer to the conduction and valence bands of Fe_2_O_3_, respectively, with no improvement toward suppression of the charge recombination. On the other hand, there are studies^19,28,34−36^ that conclude that a type II heterojunction is created, where electrons formed under visible light in Fe_2_O_3_ can be transferred to the CB of TiO_2_.^39^ However, there are also reports^4,29,37,38^ in which it is accepted that although the TiO_2_@Fe_2_O_3_ composite forms type I heterojunctions, it behaves favorably with respect to electron transfer. It is claimed that in CB_Fe_2_O_3__, higher levels exist to which the electrons can be transported. Higher levels in iron III oxide are located above CB_TiO_2__, so excited e^–^ can be injected to titanium dioxide. It should be mentioned that the type of band alignment in TiO_2_@Fe_2_O_3_ has not been elucidated based on the direct experiments concerning the heterostructures. UV–vis spectroscopy, VB X-ray photoemission spectroscopy (XPS), and work function measurements as well as photocatalysis have been carried out individually on TiO_2_ and Fe_2_O_3_. Therefore, the knowledge of the relative positions of the valence and conduction bands of these two materials is not explicitly supported by the experimental results.^30,35,38^

Most of the studies^2−6^ on the photocatalytic behavior of heterostructures aim to improve the photodegradation rate. For example, Xia et al.^2^ studied core–shell α-Fe_2_O_3_@TiO_2_ nanocomposites prepared by the heteroepitaxial growth route and showed their improved photocatalytic activity toward the decomposition of rhodamine B (RhB) in the visible light region. Yao et al.^3^ have designed and fabricated Fe_2_O_3_–TiO_2_ core–shell nanorod arrays using the glancing angle deposition technique (GLAD). These arrays have been shown to be more efficient for the degradation of methylene blue and the conversion of CO_2_ under visible light illumination. Li et al.^4^ synthesized dendritic α-Fe_2_O_3_/TiO_2_ nanocomposites for visible light degradation of eosin red, Congo red, methylene blue, and methyl orange. Huang et al.^29^ demonstrated enhanced photocatalytic denitrification of pyridine over TiO_2_/α-Fe_2_O_3_ nanocomposites under visible light irradiation. Mendiola-Alvarez et al.^5^ proposed a new P-doped Fe_2_O_3_–TiO_2_ mixed oxide prepared by a microwave-assisted sol gel method for the photocatalytic degradation of sulfamethazine (SMTZ) with better efficiency within the visible range of the electromagnetic spectrum than that of unmodified Fe_2_O_3_–TiO_2_ and TiO_2_. Wannapop et al.^6^ studied the photocatalytic degradation of RhB on 1D TiO_2_ nanorods synthesized by the hydrothermal method and decorated with Fe_2_O_3_. The level of degradation after 5 h increased from 30% for TiO_2_ to 63% for the Fe_2_O_3_/TiO_2_ heterostructure due to favorable charge transfer at the interface.

However, improvement in the photocatalytic activity is only the secondary aim of our current research. Determination of the type of the electronic structure of TiO_2_@Fe_2_O_3_ should be considered as the primary motivation for this work. The novelty is based on the particular approach to this task, which consists in the application of photocatalysis with specific scavengers of OH^•^, h^•^, e^–^, and ^•^O_2_^–^ as an experimental tool to draw conclusions regarding the CB and VB edge configuration.

In our previous paper,^33^ we have proposed the formation of an intermediate layer of TiO_2_:Fe as a consequence of Fe_2_O_3_ deposition on the surface of the TiO_2_ nanocrystal. The incorporation of Fe^3+^ ions into the TiO_2_ lattice is associated with the appearance of an additional acceptor level within the TiO_2_ band gap.

In this work, the interface of a specific morphology has been engineered, and the correlation between morphological properties and electronic structure has been demonstrated for the first time. Direct measurements, such as optical spectroscopy, XPS, scanning electron microscopy (SEM), and high-resolution transmission electron microscopy (HRTEM), allowed us to draw conclusions regarding the electronic structure of the interface and its morphology. In addition, indirect studies based on the decomposition of the classical RhB model dye by TiO_2_@Fe_2_O_3_ nanocrystals with and without selected scavengers of various active species have been carried out. A logical scheme has been proposed to eliminate the least probable decomposition routes. Knowledge of the possible mechanism of decomposition of a specific dye is believed to assist in drawing conclusions regarding the charge transfer mechanism at the TiO_2_@Fe_2_O_3_ interface.

## 2 Experimental Section

### 2.1 Synthesis of TiO_2_ Nanocrystals

A detailed description of the growth process of anatase nanocrystals has been presented in our previous article.^33^ Briefly, the hydrothermal method was used to synthesize TiO_2_ nanocrystals as a mixture of cubes and rods. Titanium tetraisopropoxide played the role of a titanium dioxide precursor, and diethanolamine acted as a shape-controlling agent. The prepared solution was heated to 215 °C for 24 h in a stainless-steel autoclave. The resulting precipitate was washed with 0.1 M HCl, distilled water, and ethanol and then dried and calcined at 500 °C for 3 h.

### 2.2 Formation of TiO_2_@Fe_2_O_3_ Heterojunctions

The preparation conditions for particular TiO_2_@Fe_2_O_3_ heterojunctions are given in Table 1. Typically, as described for the TiO_2_@2%Fe_2_O_3_ sample, 75 ml of ammonium carbonate was poured into the beaker containing 0.75 g of TiO_2_ anatase nanocrystals. During continuous stirring, 25.95 mL of iron(III) nitrate was added dropwise. Then, the temperature of the solution was increased to 70 °C to decompose ammonium carbonate into NH_3_, CO_2_, and H_2_O. After 4 h, the beaker was covered with a watch glass and placed in the dryer for 18 h at 70 °C to complete the decomposition process. The ammonia formed during heating caused the pH of the mixture to increase, and an alkaline environment was obtained, resulting in the precipitation of iron(III) hydroxide Fe(OH)_3_. The nanopowder was then collected by centrifugation and washed five times with a 0.5 %vol ammonia solution. The freshly prepared nanopowder was dispersed in isopropyl alcohol and dried at 70 °C for complete alcohol evaporation. To transform Fe(OH)_3_ deposited on the TiO_2_ surface into Fe_2_O_3_, it was necessary to carry out the calcination process at 500 °C for 2 h.

**Table 1:** Detailed Conditions of Material Preparation

		Fe/(Fe + Ti) at. ratio [%]	
sample	Fe(NO_3_)_3_^a^/TiO_2_ ratio ±0.2 [mL/g]	assumed	from EDX analysis
TiO_2_			
TiO_2_@0.2%Fe_2_O_3_	3.46	0.239	0.61(2)
TiO_2_@1%Fe_2_O_3_	15.57	1.066	1.23(2)
TiO_2_@2%Fe_2_O_3_	34.60	2.339	3.28(2) (4.09(3))^b^
TiO_2_@10%Fe_2_O_3_	155.70	9.727	10.81(4)
TiO_2_@20%Fe_2_O_3_	346.00	19.320	25.49(4) *(24.40(3))*^b^

a Fe(NO_3_)_3_ concentration: 8.665(4)·10^–3^ M, (NH_4_)_2_CO_3_—saturated solution—100 mL per 1.000(1) g of TiO_2_.

b The atomic Fe/(Fe + Ti) ratio [%] based on the XPS analysis.

### 2.3 Characterization

X-ray diffraction (XRD) was used to study the crystal structure of the obtained materials. Measurements were carried out within the 2θ range from 20 to 80° using an X’PertPro PANalytical diffractometer (Philips) equipped with a copper anode as a radiation source (K_α1_ = 0.15406 nm). The HighScore Plus software and the PDF-2 database were applied for qualitative analysis. Quantitative analysis was performed using the Rietveld method. Supplementary conclusions concerning the phase structure were drawn on the basis of Raman spectroscopy. The Jobin-Yvon LabRam HR800 spectrometer, featuring a green laser (532 nm) and a diffraction grating of 1800 g/mm, was applied. The spectra were collected in a range of 1/λ from 80 to 800 cm^–1^. A scanning electron microscope (Nova NanoSEM 200) equipped with an energy-dispersive X-ray (EDX) detector was used to calculate the Fe/(Fe + Ti) concentration. Furthermore, transmission electron microscopy (TEM) and HRTEM images were obtained using JEOL JEM-1011 and FEI Tecnai microscopes at accelerating voltages of 100 kV and 200 kV, respectively. Bright-field and high-angle annular dark-field scanning transmission electron microscopy (HAADF STEM) images were obtained in conjunction with EDX spectrum mapping to gain information on the microstructure of the TiO_2_ and TiO_2_@Fe_2_O_3_ materials. Digital Micrograph software was employed for analyzing the HRTEM images using fast Fourier transform (FFT) and inverse fast Fourier transform (IFFT) techniques, which allowed us to calculate the interplanar spacing of the observed phases.

The optical properties were determined from the total reflectance spectra *R*_tot_(λ) recorded within the wavelength range of 220–2200 nm using the JASCO V-670 UV–VIS–NIR spectrophotometer equipped with an integrating sphere of 150 nm diameter. The energies of the optical transitions were established as corresponding to the maxima of a wavelength derivative d*R*_tot_/dλ of the total reflectance coefficient with an uncertainty of 0.02 eV.

The chemical composition and electronic state of the ions at the surface were determined by XPS using a VSW spectrometer (Vacuum Systems Workshop Ltd.) with Al Kα radiation. The atomic ratio *C*_x_ of an element x on the surface was calculated as  where *A*_x_ represents the peak area of element x and *S*_x_ is the normalized sensitivity for photoelectrons (*S*_Ti_ = 4.95 and *S*_Fe_ = 10.86).

### 2.4 Photocatalytic Activity

The photocatalytic activity toward the decomposition of RhB was studied under visible light (12 Philips TL 8W/54–7656 bulb lamps) for all materials obtained. Under typical conditions, 0.075 g of the photocatalyst was dispersed in 50 mL of RhB solution (5 × 10^–5^ M). In some experiments, 1 mL of H_2_O_2_ (30%) was added to the solution and subjected to 30 min of stirring in the dark to achieve an equilibrium of adsorption–desorption, and 2 ml of the solution was collected and filtered. After a given time interval, other portions of the previously illuminated solution were removed and filtered. The UV–vis–NIR spectrophotometer, JASCO V-670, was used to measure the absorbance of the samples over the range 400–800 nm. Finally, the *C*/*C*_o_ ratio was calculated, where *C* is the concentration of RhB after a certain time of photocatalysis and *C*_o_ is the initial concentration of RhB determined for a wavelength equal to 554 nm.

To investigate the active species generated in the photocatalytic system (PS) consisting of a photocatalyst, RhB, and H_2_O_2_, scavenger experiments were performed. Ethylenediaminetetraacetic acid (EDTA-2Na, 10 mM), benzoquinone (p-BQ, 1 mM), AgNO_3_ (100 mM), and *tert*-butyl alcohol (t-BuOH) (1:20 vol.) were used as scavengers introduced into the PS in the amount of 1 ml to capture holes (h^•^), superoxide radicals (^•^O_2_^–^), electrons (e^–^), and hydroxyl radicals (OH^•^), respectively.

Tests of cyclic photocatalysis were carried out using TiO_2_@0.2%Fe_2_O_3_ and TiO_2_@2%Fe_2_O_3_ heterostructures. After dark adsorption, a 90 min photocatalytic decomposition process of RhB was carried out and repeated four times. After each decomposition process, the photocatalyst was separated from the solution of RhB by centrifugation and washed with ethanol three times. After that, the powder was dried for 4 h at 70 °C and used again.

## 3 Results

### 3.1 Crystal Structure and Morphology

**Figure 1:**
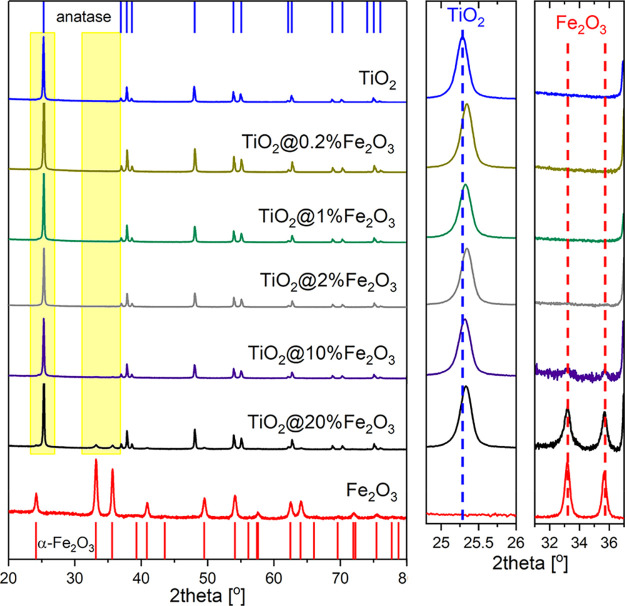
XRD patterns of TiO_2_ nanocrystals and TiO_2_ nanocrystals covered with Fe_2_O_3_. The top bars represent the positions of the anatase peaks, while the bottom bars correspond to α-Fe_2_O_3_.

The presence of iron in the heterostructures was confirmed by EDX analysis (Figure S1a), and the iron contents are shown in Table 1. The crystal structure has been identified on the basis of XRD data and the use of the PDF-2 database. Analysis has shown that TiO_2_ nanocrystals are single-phase and crystallize in a structure of anatase (JCPDS-ICDD #03-065-5714) (Figure 1) or contain traces of rutile (Figure S1b). In the case of Fe_2_O_3_, all peaks have been assigned to hematite α-Fe_2_O_3_ (JCPDS-ICDD #01-073-2234). For TiO_2_@Fe_2_O_3_, the presence of secondary phase α-Fe_2_O_3_ has been confirmed only at the highest concentration of Fe^3+^ ions during preparation. At lower concentrations of Fe^3+^ ions, XRD has not revealed any evidence for the crystallization of iron oxides. Representative XRD patterns are included in Figure 1.

Depending on the electronegativity and ionic radius, metal ions can build into oxides either interstitionally or substitutionally. The ionic radius of the Fe^3+^ ion is equal to 0.064 nm and is slightly smaller than that of Ti^4+^ ion—0.068 nm, while the Pauling electronegativities of Fe^3+^ (1.83) and Ti^4+^ (1.54) are similar. It is, thus, likely that Fe^3+^ ions substitutionally occupy cationic Ti^4+^ sites in the TiO_2_ lattice.^40^ A distortion of the lattice would manifest itself by a decrease in the lattice parameters. As a result, the positions of all TiO_2_:Fe diffraction peaks should shift to higher diffraction angles. This effect can be observed in the XRD pattern of TiO_2_@20%Fe_2_O_3_ compared to that of TiO_2_. On the basis of Rietveld analysis, the parameters of the unit cell have been calculated and found to be equal to *a* = *b* = 0.3791(1) nm, *c* = 0.9515(1) nm for TiO_2_ and *a* = *b* = 0.3785(1) nm, *c* = 0.9507(1) nm for TiO_2_@20%Fe_2_O_3_. The relative decrease in lattice parameter *a* is equal to 0.16% while that of parameter *c* is about 0.08%. This change indicates the incorporation of Fe^3+^ ions into the cationic sublattice of anatase.^33^

**Figure 2:**
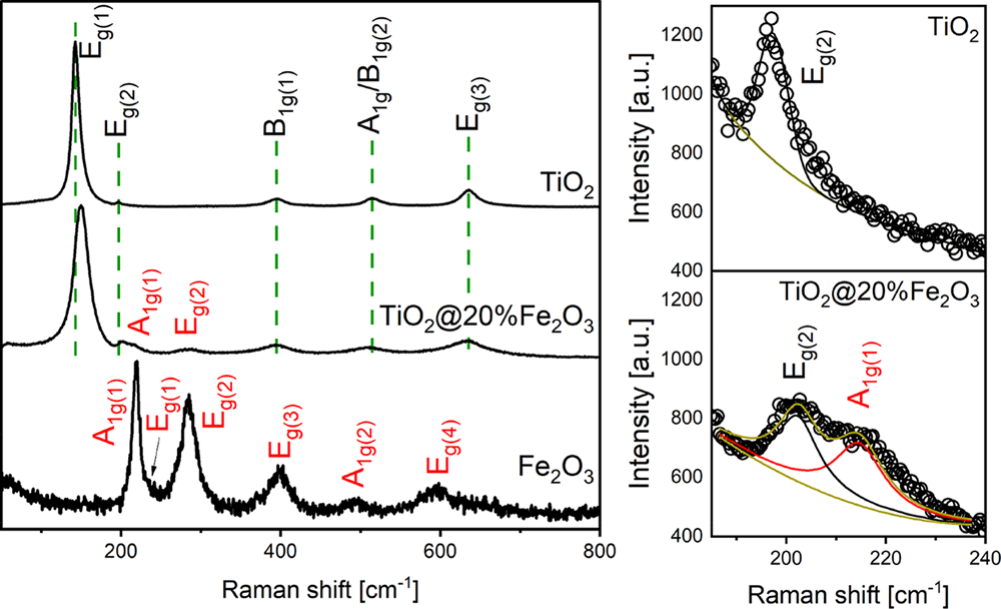
Raman spectra of TiO_2_, TiO_2_@Fe_2_O_3_, and Fe_2_O_3_ nanomaterials.

The Raman spectra of the TiO_2_, TiO_2_@Fe_2_O_3_, and Fe_2_O_3_ nanocrystals are shown in Figure 2. A typical spectrum composed of five bands is observed for anatase TiO_2_, while that of α-Fe_2_O_3_ contains six well-developed bands.^41−43^ The positions of all Raman peaks are given in Table 2.

**Table 2:** Raman Shift and Intensity of the Selected Bands for TiO_2_, Fe_2_O_3_, and TiO_2_@20%Fe_2_O_3_ Nanomaterials

	E_g(1)_ of TiO_2_		A_1g(1)_ of Fe_2_O_3_		E_g(2)_ of Fe_2_O_3_	
sample	Raman shift [cm^–1^]	intensity [a.u.]	Raman shift [cm^–1^]	intensity [a.u.]	Raman shift [cm^–1^]	intensity [a.u.]
TiO_2_	143(1)	29041(5)				
TiO_2_@20%Fe_2_O_3_	149(1)	6026(1)	214(1)	211(1)	284(1)	134(1)
Fe_2_O_3_			218(1)	177(1)	284(1)	131(1)

When considering TiO_2_@Fe_2_O_3_, in addition to the bands that can be attributed to anatase, two surplus bands can be seen. The Raman shift corresponding to 213–223 cm^–1^ is attributed to α-Fe_2_O_3_ A_1g(1)_.^42,43^ Another band appearing at 284 cm^–1^ can be attributed α-Fe_2_O_3_ E_g(2)_.^41−43^ Substitutional incorporation of Fe^+3^ ions for Ti^+4^ cations, as determined by XRD data, is further supported by the change of the E_g(1)_ band from 143 cm^–1^ (TiO_2_ nanocrystals) to 149 cm^–1^ (TiO_2_@20%Fe_2_O_3_) with simultaneous reduction of band intensity by 79%, as shown in Table 2. It has been suggested^44^ that the shift in the anatase E_g(1)_ Raman band is the result of lattice defects. TiO_2_ doping with iron(III) ions was postulated in our previous paper^33^ based on the spectrophotometric data.

TEM images provided information on the shape and size of the particles (Figure S2). Titanium dioxide nanocrystals form elongated rods ca. 230 nm long (Figure S2a). The analysis of TiO_2_@Fe_2_O_3_ indicates that Fe_2_O_3_ forms a discontinuous layer of nanograins with a size of 15–50 nm at the surface of the TiO_2_ nanocrystals (Figure S2b,c). In the case of TiO_2_@2%Fe_2_O_3_, Fe_2_O_3_ crystals take a needle-like shape and grow only on certain walls of TiO_2_ (Figure S2b).

**Figure 3:**
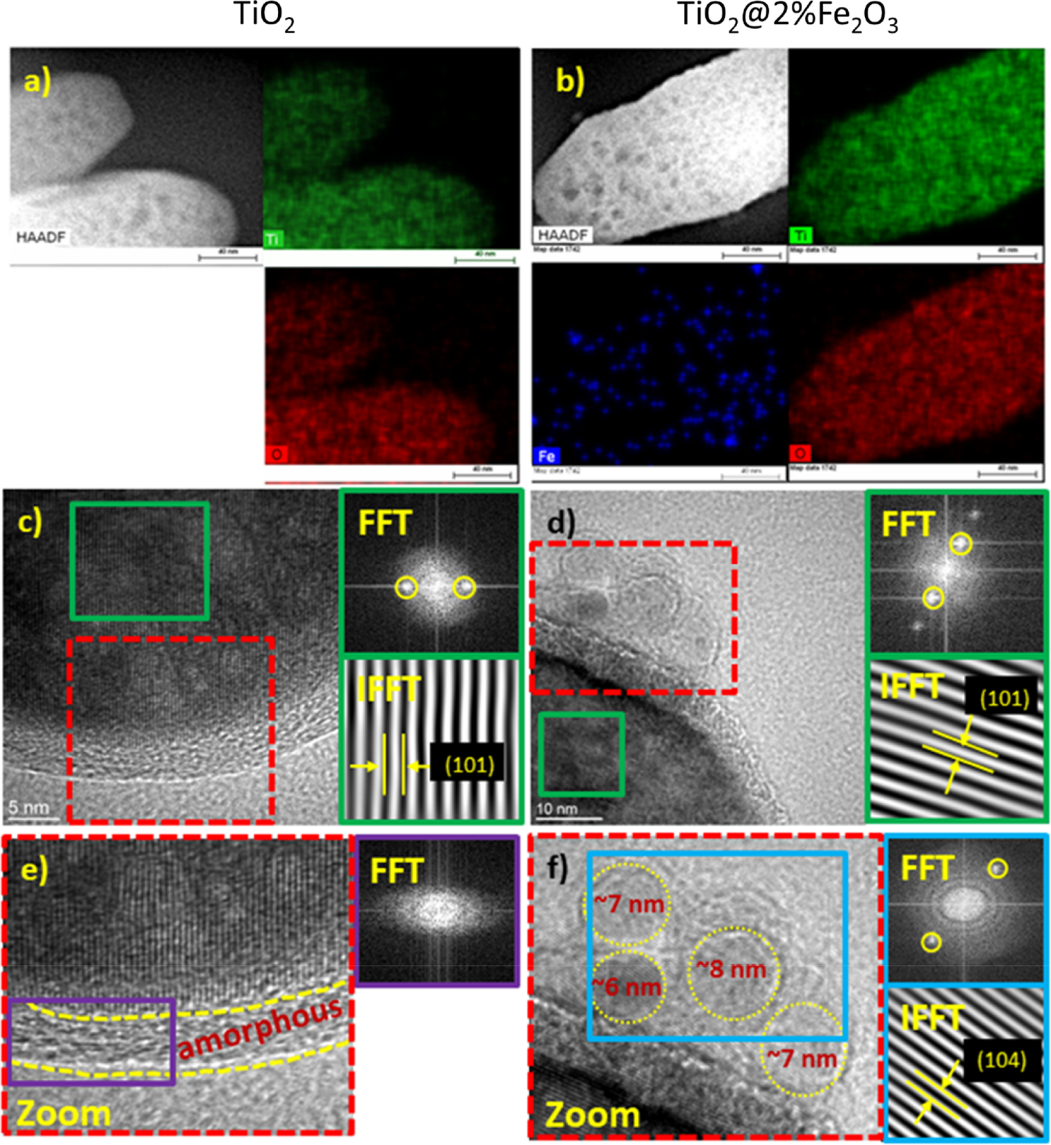
(a,b) EDX mapping images; (c,d) HRTEM images with FFT and IFFT analyses indicate the existence of the (101) plane of anatase TiO_2_; (e,f) FFT of the purple/blue rectangle of the zoomed-in area shows an amorphous coating (TiO_2_) or hematite nanoparticles of size 6–8 nm in the (104) plane of α-Fe_2_O_3_ (TiO_2_@2%Fe_2_O_3_).

EDX mapping and analysis of HRTEM images are shown in Figure 3. All IFFT calculations were performed after noise reduction using spots marked with a yellow circle in the FFT patterns. Then, masking was applied, and the resulting IFFT images presented the arrangement of crystallographic planes (black lines), which allowed the measurement of interplanar spacing.

EDX spectrum mapping of the regions shown in HAADF STEM images (Figure 3a,b) was used to create maps of Ti, O, and Fe elements. These results reveal that in the case of TiO_2_@2%Fe_2_O_3_, iron is distributed homogeneously (Figure 3b), and for the TiO_2_@20%Fe_2_O_3_ sample, Fe_2_O_3_ grains of size tens of nanometers are also observed (Figure S3a).

Well-crystallized structures can be observed for all synthesized powders that are represented by the distinct spots on the FFT patterns. The green rectangles in Figures 3c,d, and S3b indicate the investigated area, the ROI (region of interest) of the FFT analysis. In each case, the spots obtained from the ROI correspond to an interplanar spacing of about 0.354 nm, which is well-correlated with the {101} plane of anatase TiO_2_, the presence of which is also confirmed by XRD studies. However, the amorphous layer that was a part of the rod was observed at the surface of the TiO_2_ nanocrystals (Figure 3e). Furthermore, in the HRTEM images of the TiO_2_@2%Fe_2_O_3_ and TiO_2_@20%Fe_2_O_3_ composites (Figures 3f and S3c), nanograins of size 6–8 nm deposited on the surface of the TiO_2_ nanocrystals were found. To investigate their crystal structure, we performed the FFT analysis from the area in the blue rectangle, and then, the reverse FFT analysis was executed. The measured interplanar spacing was equal to 0.273 nm, which is close to the 0.270 nm lattice spacing of the {104} crystal planes of hematite.

### 3.2 Electronic Structure

The electronic structure of the components of the composite materials, such as TiO_2_@Fe_2_O_3_, studied by XPS and optical spectrophotometry, plays a special role in the prediction of the character and type of the interface. Additional information about the interfacial charge transfer processes can be obtained from the carefully designed photocatalytic experiment. In this work, we have carried out a series of tests aimed at the photocatalytic degradation of the standard dye, that is, RhB, with and without certain scavengers. From the rates of decomposition of dye, conclusions about the probabilities of charge transfer processes can be drawn, which helps figure out the electronic structure of the interface.

**Figure 4:**
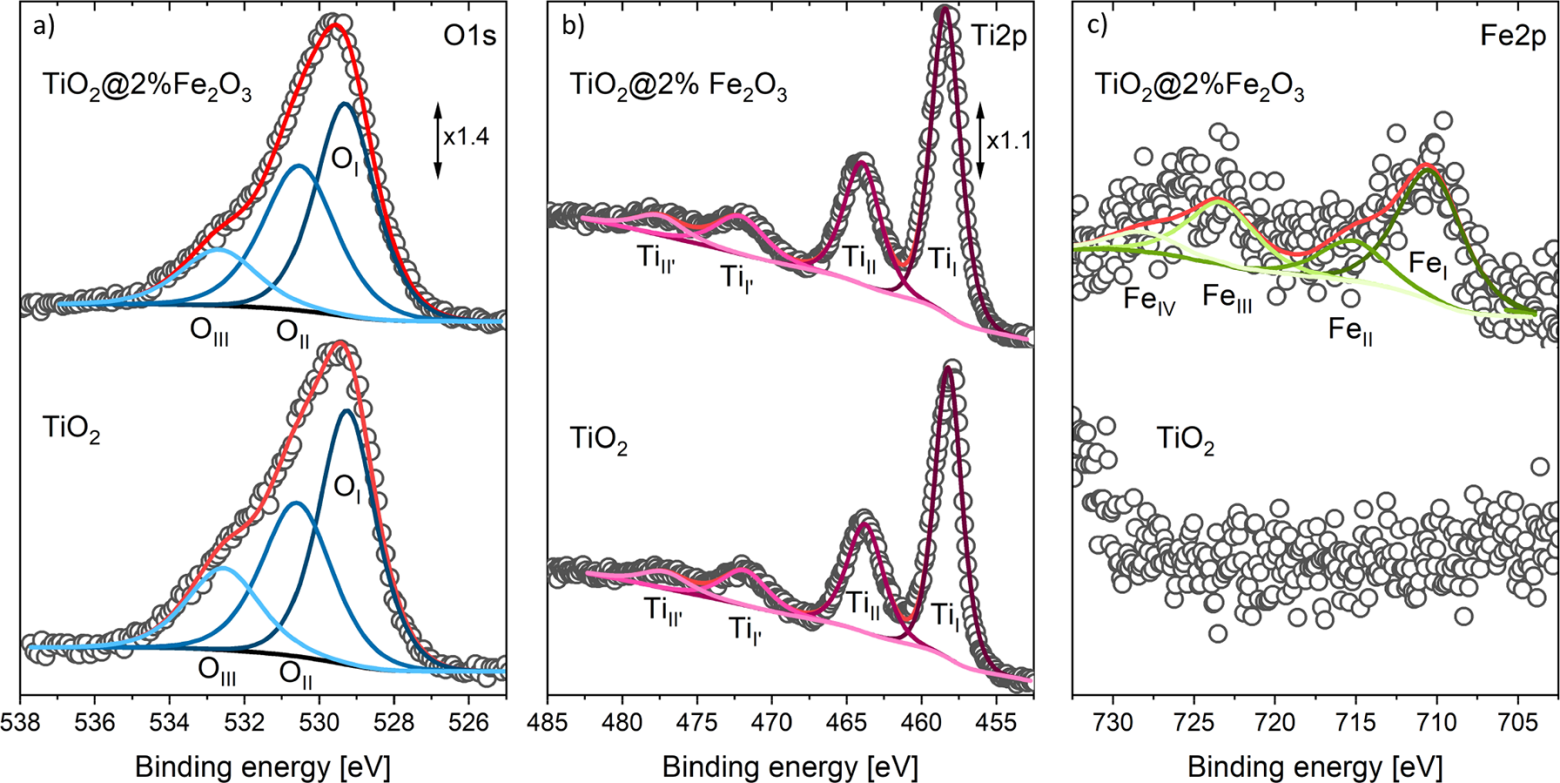
Deconvolution of the O1s, Ti2p, and Fe2p XPS spectra of TiO_2_ and TiO_2_@2%Fe_2_O_3_ nanocrystals.

Surface chemistry of the selected elements: Ti, O, and Fe, as well as surface defects, were studied by means of XPS. Figure 4 shows the high-resolution spectra of O1s, Ti2p, and Fe2p from the TiO_2_ and TiO_2_@2%Fe_2_O_3_ nanocrystals. XPS data concerning TiO_2_@20%Fe_2_O_3_ nanocrystals with the highest amount of Fe_2_O_3_ are presented in Figure S4. The values of all binding energies determined by the fitting of the different XPS lines are given in Tables 3 and S1.

**Table 3:** Results of XPS Analysis of TiO_2_ and TiO_2_@2%Fe_2_O_3_ Nanocrystals—Assignment of Ti2p, O1s, and Fe2p peaks to Certain Types of Bonding

	binding energy (eV)			
symbol	TiO_2_	TiO_2_@2%Fe_2_O_3_	type of bonding	refs
Ti2p				
Ti_I_	458.2(3)	458.4(3)	Ti 2p_3/2_, O–Ti–O	[^45^]
Ti_II_	463.8(3)	464.0(3)	Ti 2p_1/2_, O–Ti–O	[^45^]
Ti_I′_	472.1(3)	472.2(3)	satellite of Ti2p_3/2_, O–Ti–O	[^45^]
TiI_I′_	477.3(3)	477.6(3)	satellite of Ti2p_1/2_, O–Ti–O	[^45^]
O1s				
O_I_	529.3(4)	529.3(4)	Ti–O–Ti	[^46^]
O_II_	530.6(4)	530.6(4)	oxygen vacancies or defects	[^46^]
O_III_	532.7(4)	532.7(4)	chemisorbed species, e.g., OH^–^, H_2_O, O^2–^	[^46^]
Fe2p				
Fe_I_		710.6(7)	Fe 2p_3/2_, Fe^3+^ in Fe_2_O_3_	[^47, 49^]
Fe_II_		715.2(8)	satellite of Fe 2p_3/2_, Fe^3+^ in Fe_2_O_3_	[^47, 49^]
Fe_III_		723.5(7)	Fe 2p_1/2_, Fe^3+^ in Fe_2_O_3_	[^47, 49^]
Fe_IV_		728.4(7)	satellite of Fe 2p_1/2_, Fe^3+^ in Fe_2_O_3_	[^47, 49^]

The shape of the Ti2p XPS peak is complex in all samples. In the case of TiO_2_, four components with the following binding energies 458.2 eV (Ti_I_), 463.8 eV (Ti_II_), 472.1 eV (Ti_I’_), and 477.3 eV (Ti_II’_) were fitted. The Ti2p_3/2_–Ti2p_1/2_ (Ti_I_–Ti_II_) doublet arises from spin orbit splitting and can be ascribed to Ti^4+^ ions in TiO_2_ (titanium–oxygen–titanium bonding). The higher energies of 472.1 eV (Ti_I’_) and 477.3 eV (Ti_II’_) correspond to the satellite peaks of Ti2p_3/2_ and Ti2p_1/2_, respectively.^45^ Upon covering TiO_2_ nanocrystals with Fe_2_O_3_, the Ti2p XPS peaks shift slightly toward higher binding energies, and their exact positions depend on the amount of deposited Fe_2_O_3_, that is, the shift of 0.1–0.3 eV is observed at the lower amount of Fe_2_O_3_ (TiO_2_@2%Fe_2_O_3_) while that of 0.6–1.0 eV at the higher amount of Fe_2_O_3_ (TiO_2_@20%Fe_2_O_3_). The shift of these peaks indicates changes in the chemical environment of Ti^4+^ ions.

Analysis of the O1s peak reveals three components at 529.3 eV (O_I_), 530.6 eV (O_II_), and 532.7 eV (O_III_). The lowest-energy O_I_ component is related to lattice oxygen O^2–^ in a fully coordinated position in the TiO_2_ lattice (titanium–oxygen–titanium bonding). The highest-energy O_III_ component is associated with species, such as hydroxyl groups OH^–^, water molecules H_2_O, or dissociated oxygen O^2–^, chemisorbed at the surface. The medium-energy O_II_ component, in agreement with reports in the literature,^46^ can be attributed to the oxygen vacancy V_O_ in the titanium dioxide lattice. It should be noted that a small amount of Fe_2_O_3_ on the surface of TiO_2_ does not cause any changes to the positions of the O1s peaks, while its high amount causes a shift of about 0.1–0.2 eV toward higher binding energies (Figure S4).

The Fe2p XPS peak has been fitted with four lines. In the case of TiO_2_@2%Fe_2_O_3_, they are located at 710.6 eV (Fe_I_), 715.2 eV (Fe_II_), 723.5 eV (Fe_III_), and 728.4 eV (Fe_IV_). Two main Fe_I_ and Fe_III_ peaks correspond to Fe2p_3/2_ and Fe2p_1/2_ states, respectively, which shows that iron is present in the form of Fe^3+^ ions. The component denoted Fe_II_ has been assigned to the satellite of Fe2p_3/2_ and to the satellite of Fe2p_1/2_.^47−49^ At the highest amount of Fe_2_O_3_, not only an increased intensity of these peaks is observed but a shift of about 0.4–0.8 eV toward higher binding energies can also be seen (Figure S4). Quantitative analysis of XPS data indicates that the atomic fraction of iron reaches 4.1% in the case of lower (TiO_2_@2%Fe_2_O_3_) and 24.4% for the highest amount of Fe_2_O_3_ (TiO_2_@20%Fe_2_O_3_) at the surface of TiO_2_ nanocrystals (Table 1).

Although the presence of iron oxide does not cause a drastic shift in the XPS peaks of O1s, it manifests itself quite differently. The deposition of Fe_2_O_3_ at the surface of TiO_2_ nanocrystals leads to changes in the area under the O1s lines. In particular, the O_II_ component at the medium binding energy is affected. An increase in the amount of Fe_2_O_3_ deposited is accompanied by an increase in the fraction attributed to oxygen vacancies. For TiO_2_ nanocrystals, the O_II_ fraction is equal to 34.4% and gradually increases to 38.2% for a low amount of Fe_2_O_3_ and up to 63.3% for a high amount of Fe_2_O_3_ on the surface of TiO_2_. The increase in V_O_ contribution combined with the fact that the presence of a large amount of Fe_2_O_3_ results in a slight shift of the peaks can be treated as a proof of substitutional doping of Fe^3+^ into the titanium sublattice. The formation of oxygen vacancies at the surface of titanium dioxide can bring two additional electrons associated with one V_O_. As a result, two Ti^3+^ ions can appear.^46^

**Figure 5:**
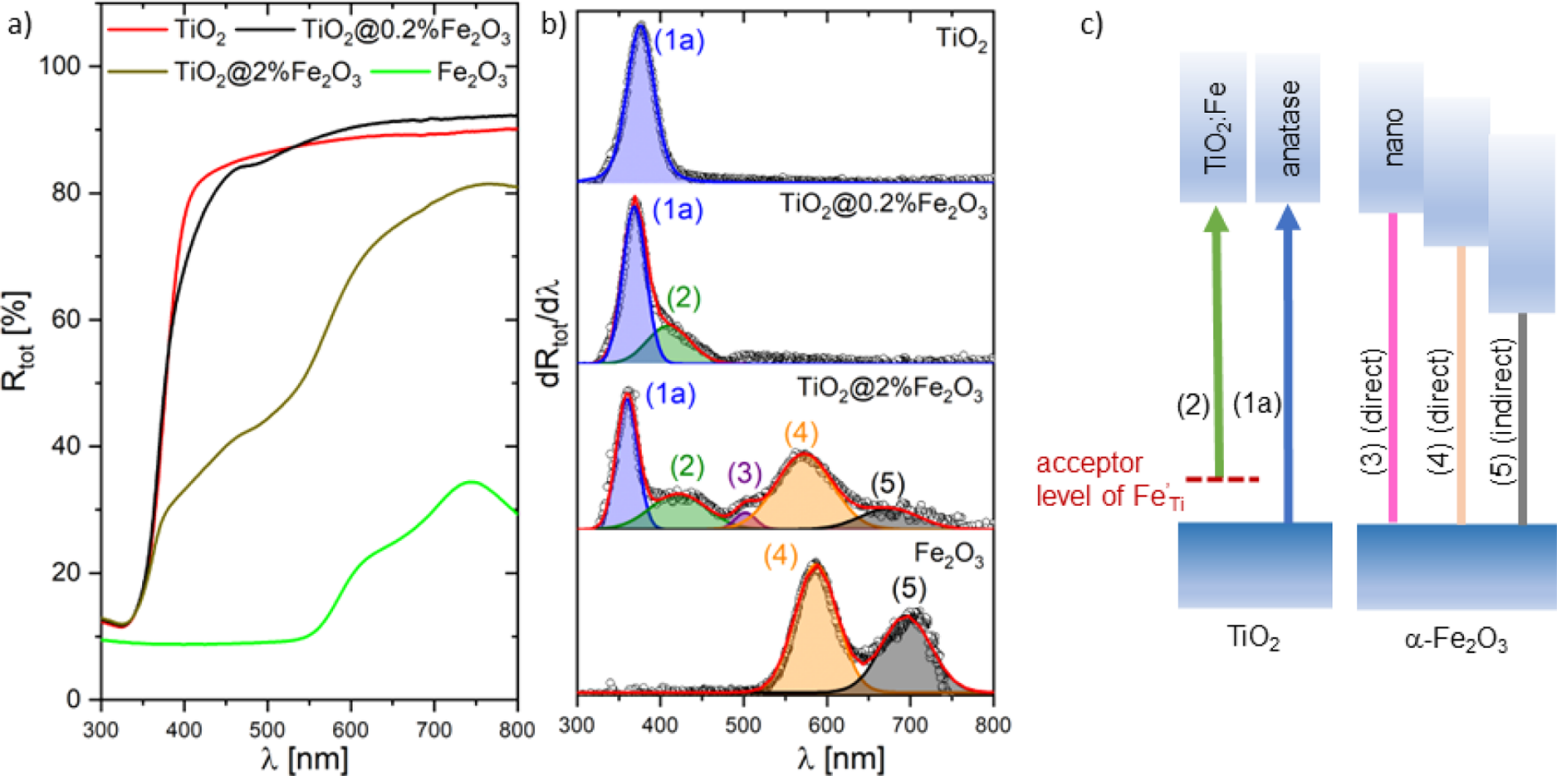
(a) Spectral dependence of the total optical reflectance *R*_tot_, (b) first derivative spectra d*R*_tot_/dλ of TiO_2_@Fe_2_O_3_; hν—photon energy, and (c) diagrams of optical transitions in TiO_2_ and α-Fe_2_O_3_.

The comparison of the optical reflectance spectra of TiO_2_ before and after being covered with Fe_2_O_3_ is presented in Figures 5a (0.2 and 2%) and S5 (1, 10, and 20%). Figures 5b and S5b demonstrate the first derivative spectra d*R*_tot_/dλ of TiO_2_, TiO_2_@Fe_2_O_3_, and Fe_2_O_3_. All possible optical transitions are listed in Table S2 and shown schematically in Figure 5c.

In the case of pure TiO_2_, the peak in d*R*_tot_/dλ can be seen at 3.32 eV, corresponding to anatase (1a). The same peak appears in d*R*_tot_/dλ of TiO_2_@2%Fe_2_O_3_ at slightly different positions 3.43 eV (1a) due to the presence of iron oxide. Furthermore, four additional transitions have been identified for TiO_2_@Fe_2_O_3_. Three of them have been assigned:

- (2) at 2.9 eV—level of Fe^3+^ impurity in the forbidden band of TiO_2_,^50,51^
- (4) at 2.2 eV—direct optical transition from the VB to CB in α-Fe_2_O_3_,
- (5) at 1.8 eV—indirect optical transition from the VB to CB in α-Fe_2_O_3_.^52^

In addition, a fourth transition (3) at 2.5 eV has been observed in this work when the Fe/(Fe + Ti) atomic ratio exceeded 1%. The origin of this transition remained unclear for a long time until it was realized that it could be attributed to the size effect in Fe_2_O_3_.^53^ For nanoparticles with a size decreasing from 120 nm to 7 nm, an increase in the band gap energy from 2.18 to 2.95 eV was demonstrated. This effect has been attributed to the size effect, which was accompanied by a modification of the hematite structure. Fondell et al.^52^ analyzed optical absorption as a function of film thickness for hematite. Two direct transitions were found at 2.15 and 2.45 eV, and they were blue-shifted by 0.3 and 0.45 eV, respectively, when the film thickness was decreased from 20 to 4 nm.

In the present work, structural studies do not show any evidence of phases other than α-Fe_2_O_3_ of iron oxide. TEM and HRTEM images for TiO_2_@2%Fe_2_O_3_ and TiO_2_@20%Fe_2_O_3_ composites (Figures 3, S2, and S4) confirm that iron oxide nanoparticles deposited at the surface of TiO_2_ nanocrystals crystallize as α-Fe_2_O_3_. The combination of SEM and HRTEM studies indicates that the applied synthesis method produces relatively large 15–50 nm as well as very small 6–8 nm α-Fe_2_O_3_ nanoparticles on the surface of TiO_2_. Therefore, we propose that the additional 2.48 eV optical transition is associated with the presence of small Fe_2_O_3_ nanoparticles.

### 3.3 Photocatalytic Degradation of RhB

**Scheme 1:**
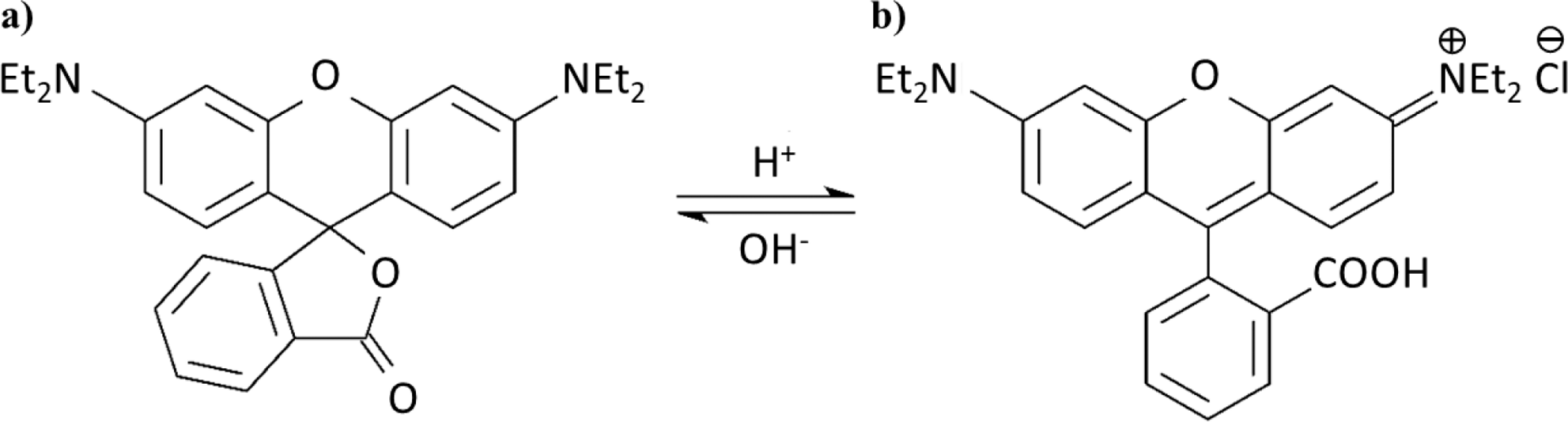
Structural Formula of RhB in (a) Open and (b) Closed Forms

RhB, as a model cationic aminoxanthene dye, is widely applied in the textile and color glass industry.^954^ Its structural formula is shown in Scheme 1.

Efficient degradation of RhB is of upmost importance due to its high toxicity. There are many degradation schemes of RhB, one of them being laser cavitation.^955^ The N-de-ethylation and chromophore cleavage are two degradation pathways of RhB.

In this paper, we have undertaken photocatalytic methods of dye decomposition. Under the illumination of RhB, electron transitions occur due to the presence of orbitals: HOMO (the highest occupied molecular orbital) and LUMO (the lowest unoccupied molecular orbital). For RhB, the HOMO level is at −4.97 eV and the LUMO level is at −2.73 eV relative to vacuum, giving an energy gap of 2.23 eV.^956^

Photodegradation of RhB can proceed via three oxidation routes^54,55^
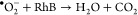
1
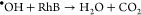
2
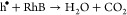
3

To decide which of these three routes will play the most important role, one must consider the availability of active radicals: ^•^O_2_^–^ and ^•^OH, as well as holes and electrons in the PS. Active species are formed in the interactions of electrons e^–^ and holes h^•^ with dissolved oxygen, water, and hydrogen peroxide, according to the following reactions:^56,57^
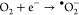
4
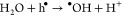
5
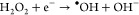
6

**Figure 6:**
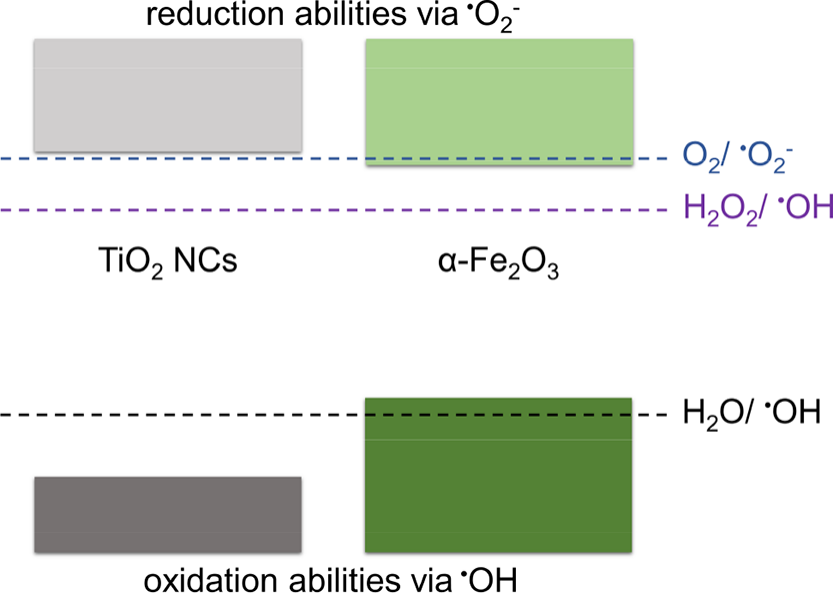
Energy band diagram of pure TiO_2_ and Fe_2_O_3_.

In PSs containing metal oxide semiconductors, electrons and holes are provided by irradiation with photons of energy hν exceeding their band gap energy (hν ≥ E_g_). Oxidation and reduction abilities of the photogenerated charge carriers depend not only on the band gap of a photocatalyst but also on the proper alignment of its conduction CB and VB edges with respect to the oxidation and reduction levels of active species, as shown in Figure 6. Therefore, from the photocatalytic performance of single semiconductors or their heterojunctions, one can draw conclusions concerning their electronic structure.

Stoichiometric TiO_2_ is a wide-band semiconductor (E_g_ = 3.0–3.2 eV) with CB and VB edges properly aligned with respect to the levels of oxidation and reduction of active radicals. Electrons photoexcited to the CB and holes left in the VB as a result of UV light absorption in TiO_2_ can participate in reactions (eq 4) and (eq 5). The reaction (eq 6) is also possible in the presence of H_2_O_2_. However, visible radiation will produce an effect only in the case of defects responsible for the introduction of additional levels in the forbidden band gap of TiO_2_.

On the other hand, the lower band gap of Fe_2_O_3_ allows visible light to generate electrons and holes. However, since CB_Fe2O3_ is located near the reduction potential of O_2_/^•^O_2_^–^ (see Figure 6), electrons created under visible light in the CB of Fe_2_O_3_ can only reduce oxygen to superoxide radicals to a small extent.

Furthermore, the process described by eq 5 cannot occur in this oxide because its VB is above the water oxidation potential H_2_O/^•^OH, while reduction of hydrogen peroxide (eq 6) is possible due to the CB edges being above the H_2_O_2_/^•^OH potential. For this reason, preliminary photocatalytic studies under visible light carried out without the addition of H_2_O_2_ showed that dye decomposition was negligible (Figure S6a,b).

The influence of H_2_O_2_ addition on RhB decomposition without a photocatalyst was also investigated in the system RhB + H_2_O_2_ + vis (Figure S6b). The results showed no negative impact of the additive, that is, no photolysis of H_2_O_2_ was observed. Therefore, the rest of the photocatalytic tests were performed with its addition.

**Figure 7:**
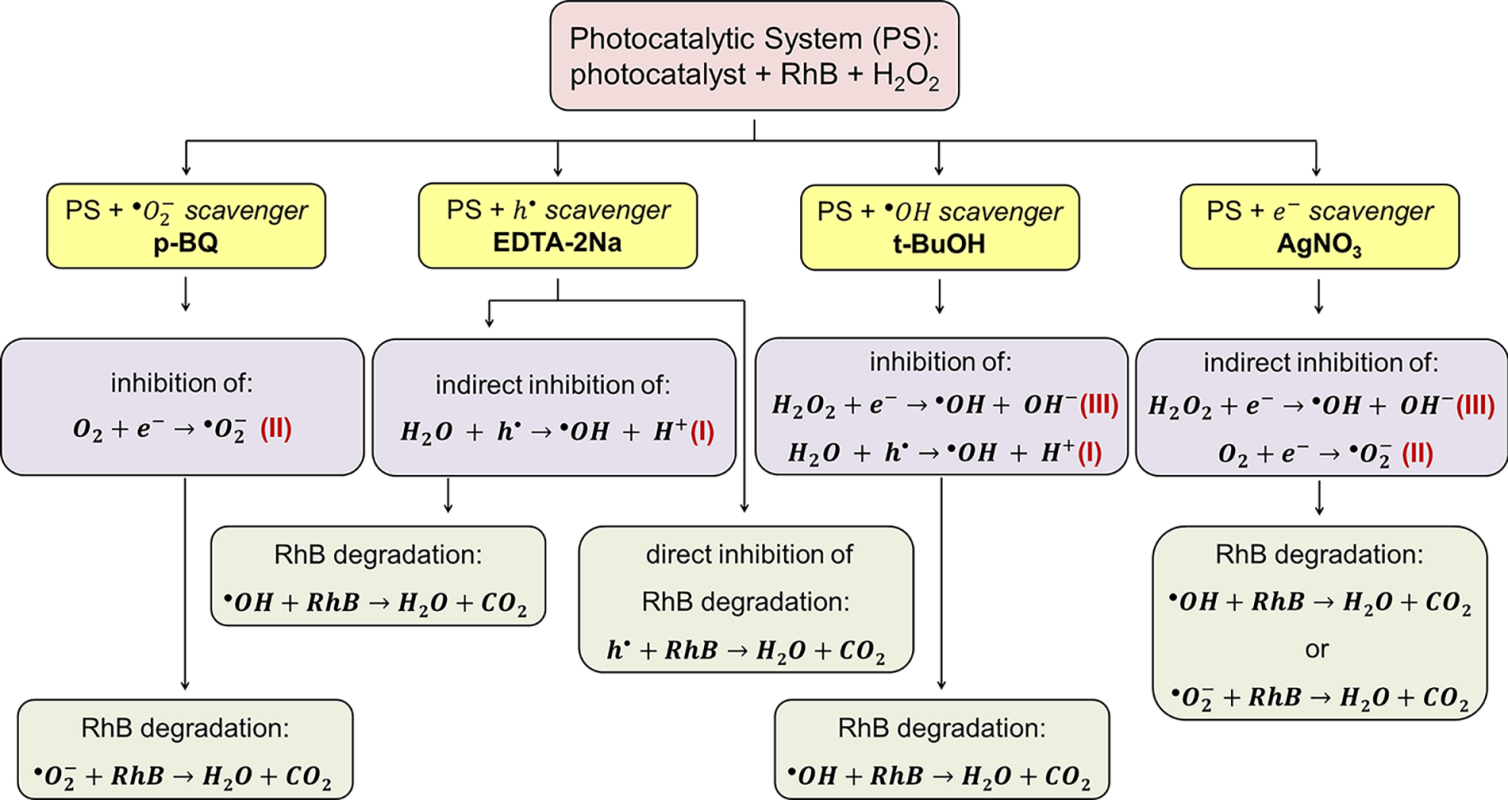
Schematic diagram showing the effect of the addition of particular scavengers on the mechanism of RhB decomposition.

The energy band diagram presented in Figure 6 corresponds to single pure photocatalysts of TiO_2_ and Fe_2_O_3_ treated separately. To construct the corresponding electronic model of the interface between TiO_2_ and Fe_2_O_3_, additional well-programed experiments with scavengers of the discussed radicals were performed, as illustrated in Figure 7. A drastic decrease in the photocatalytic activity after the addition of a specific scavenger indicates that the captured radicals are the main active species in the PS. In this paper, tert-butyl alcohol (t-BuOH), disodiumethylene diaminetetraacetate (EDTA-2Na), *p*-benzoquinone (p-BQ), and AgNO_3_ were used as ^•^OH, h^•^, ^•^O_2_^–^, and e^–^ scavengers, respectively.

**Figure 8:**
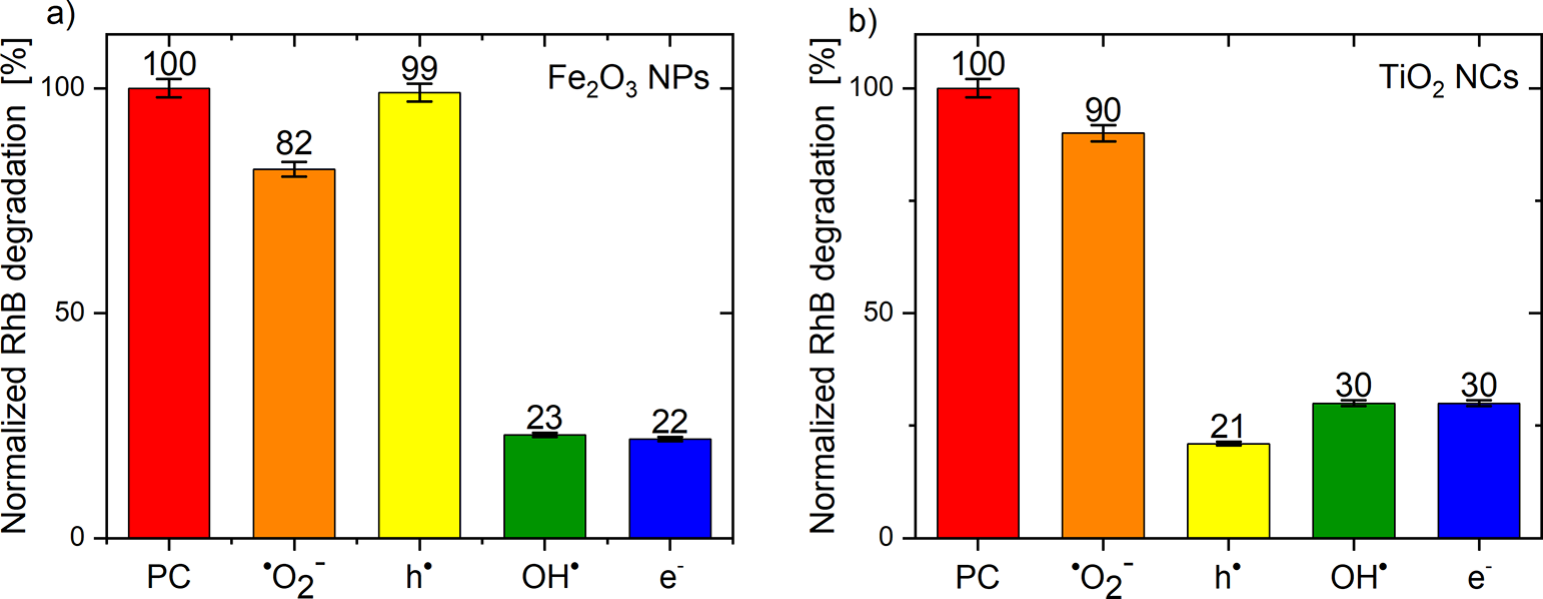
Effect of radical scavengers on RhB photocatalytic degradation on Fe_2_O_32_ (a) and TiO_2_ (b) photocatalysts under visible light.

The influence of scavengers on RhB decomposition is quite different for pure TiO_2_ and Fe_2_O_3_. Figure 8 shows normalized dye degradation after 60 min from the addition of a specific scavenger in relation to degradation without its addition
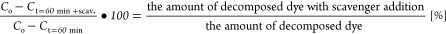
7

**Figure 9:**
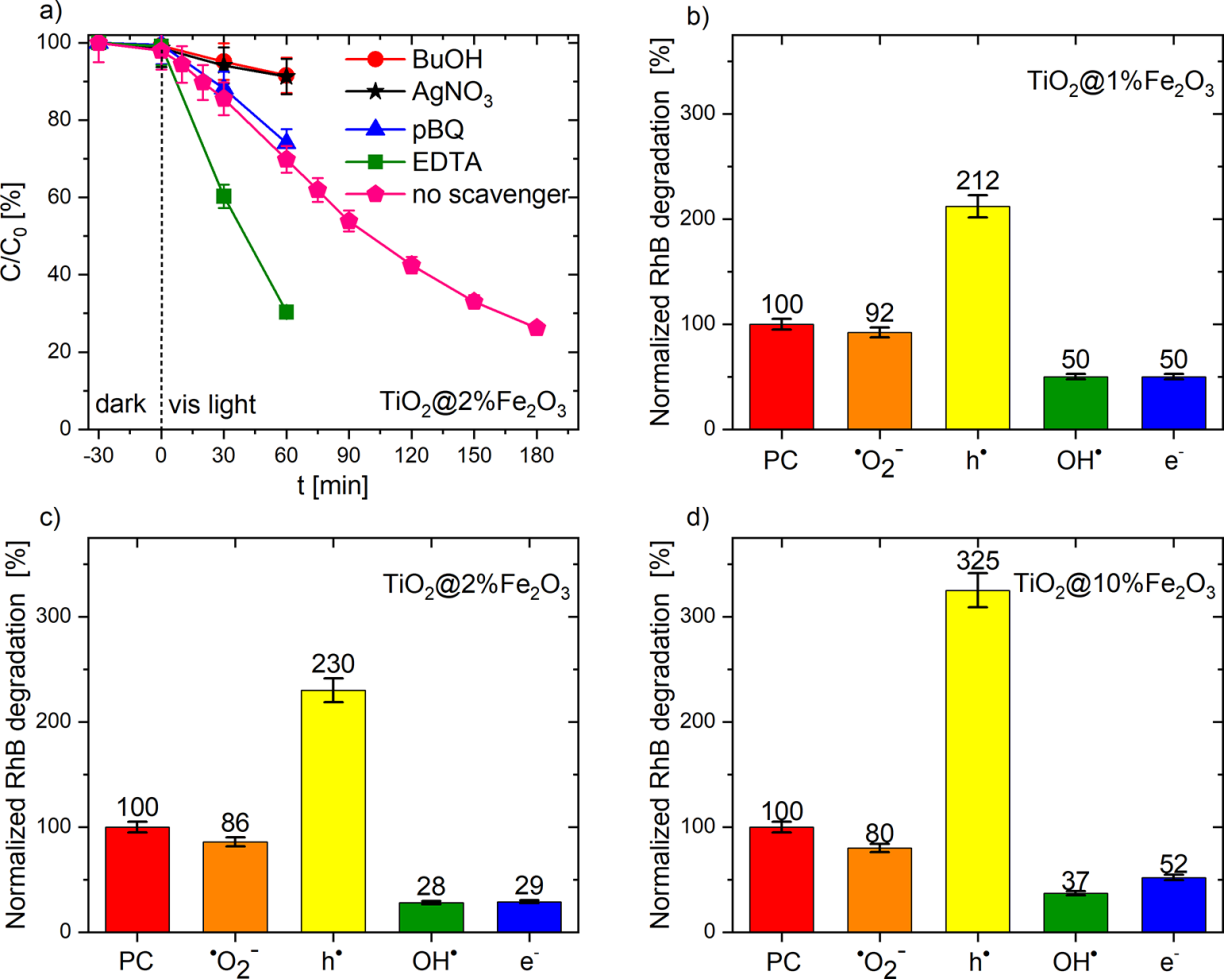
Effect of radical scavengers on RhB photocatalytic degradation on TiO_2_@Fe_2_O_3_ photocatalysts under visible light: example kinetics of the dye decomposition on TiO_2_@2%Fe_2_O_3_ (a) and normalized degradation of the dye on TiO_2_@1%Fe_2_O_3_ (b), TiO_2_@2%Fe_2_O_3_ (c), and TiO_2_@10%Fe_2_O_3_ (d).

The abbreviation PC in Figures 8 and 9 denotes a normalized dye degradation without the addition of scavengers; therefore, it is always equal 100%. The photocatalytic activity of hematite nanoparticles does not change after the addition of a hole scavenger (EDTA-2Na) (Figure 8a), which is consistent with the statement discussed previously (Figure 6) that VB_Fe2O_3__ is above the oxidation potential of water (reaction I does not occur, see Figure 7). The abbreviation PC in Figures 8 and 9 denotes a normalized dye degradation without the addition of scavengers; therefore, it is always equal 100%. The photocatalytic activity of hematite nanoparticles does not change after the addition of a hole scavenger (EDTA-2Na) (Figure 8a), which is consistent with the statement discussed previously (Figure 6) that VB_Fe2O_3__ is above the oxidation potential of water (reaction I does not occur, see Figure 7). Furthermore, superoxide radicals have been shown to play a minor role in RhB decomposition as CB_Fe2O3_ is located only slightly higher than the O_2_/^•^O_2_^–^ potential, which does not allow electrons to completely reduce the dissolved oxygen in the dye solution (reaction II, see Figure 7). On the other hand, a high decrease in photoactivity is observed when e^–^ and OH^•^ are captured from the system. This shows that OH^•^ radicals, which are formed in reaction III, play a key role in the decomposition of RhB dye (see Figure 7). After the electrons are scavenged, the reduction of H_2_O_2_ to OH^•^ is stopped, and therefore, the photocatalytic process is slower.

Different situations occur when we consider TiO_2_ nanocrystals (Figure 8b). In this case, a decrease in the photocatalytic activity is observed after the addition of the e^–^, h^•^, and OH^•^ scavengers. This means that the main active species, as in the case of Fe_2_O_3_, are hydroxyl radicals, but originating from two different reactions. First, there is the reduction of hydrogen peroxide by electrons (reaction III, see Figure 7), and second, water is oxidized by holes (reaction I, see Figure 7). Moreover, the addition of the ^•^O_2_^–^ scavenger has little effect on photocatalysis as CB_TiO2_ is located closely to the O_2_/^•^O_2_^–^ potential (Figure 6).

Additional changes occur in the case of a heterojunction composed of anatase nanocrystals covered with iron oxide nanoparticles (Figure 9). The p-BQ (^•^O_2_^–^) scavenger slightly reduces the photoactivity of the tested materials. This means that in this case as well, superoxide radicals play a minor role in RhB photodegradation (Figure 9b–d). On the other hand, after scavenging the OH^•^ radicals and electrons from the system, a significant decrease in RhB decomposition was observed (inhibition of reaction III, see Figure 7) because the hydroxyl radicals from H_2_O_2_ reduction are the main active species in the decomposition of RhB. However, the most interesting effect was observed after the addition of a hole scavenger. It is assumed that the elimination of h^•^ from the system reduces the recombination rate, and thus, more electrons were able to reduce H_2_O_2_. Furthermore, it should be noted that the increase in photocatalytic activity after the addition of EDTA-2Na (h^•^) is proportional to the amount of Fe_2_O_3_ in the heterojunction (1%—212, 2%—230, and 10%—325). The small amount of hematite (TiO_2_@1%Fe_2_O_3_) is responsible for the low area of the TiO_2_@Fe_2_O_3_ interface where recombination can occur. When this surface increases, the number of probable recombination sites also increases; therefore, effective scavenging of holes resulted in a high increase in photoactivity in the case of TiO_2_@10%Fe_2_O_3_ (Figures 9d and S7).

As the stability of the photocatalysts is a very important issue for practical applications, the recyclability photocatalytic tests were performed. Two selected heterostructures were subjected to the RhB photodegradation in a sequence of four successive reactions (Figure S8). After the first cycle, the efficiency of photocatalysts decreases slightly; however, in the third and fourth cycles, it remains constant. This allows us to conclude that the obtained TiO_2_@Fe_2_O_3_ heterostructures show stability in the cyclic photocatalytic process.

## 4 Discussion

Analysis of the spectral dependence of the absorption coefficient presented in Figures 5 and S5 shows that the presence of iron oxide Fe_2_O_3_ strongly modifies its shape and moves the fundamental absorption edge from UV toward the visible range. The characteristic energies of the optical transitions in TiO_2_, Fe_2_O_3_, and TiO_2_@Fe_2_O_3_ determined as the maxima in the first derivative of d*R*_tot_/dλ are given in Table S2. The trivalent iron metal dopants Fe^3+^ can act as acceptors. The incorporation of Fe^3+^ into TiO_2_ with an ionic radius (0.064 nm) smaller than that of Ti^4+^ (0.068 nm) can be expressed by the following reaction
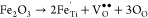
8

**Figure 10:**
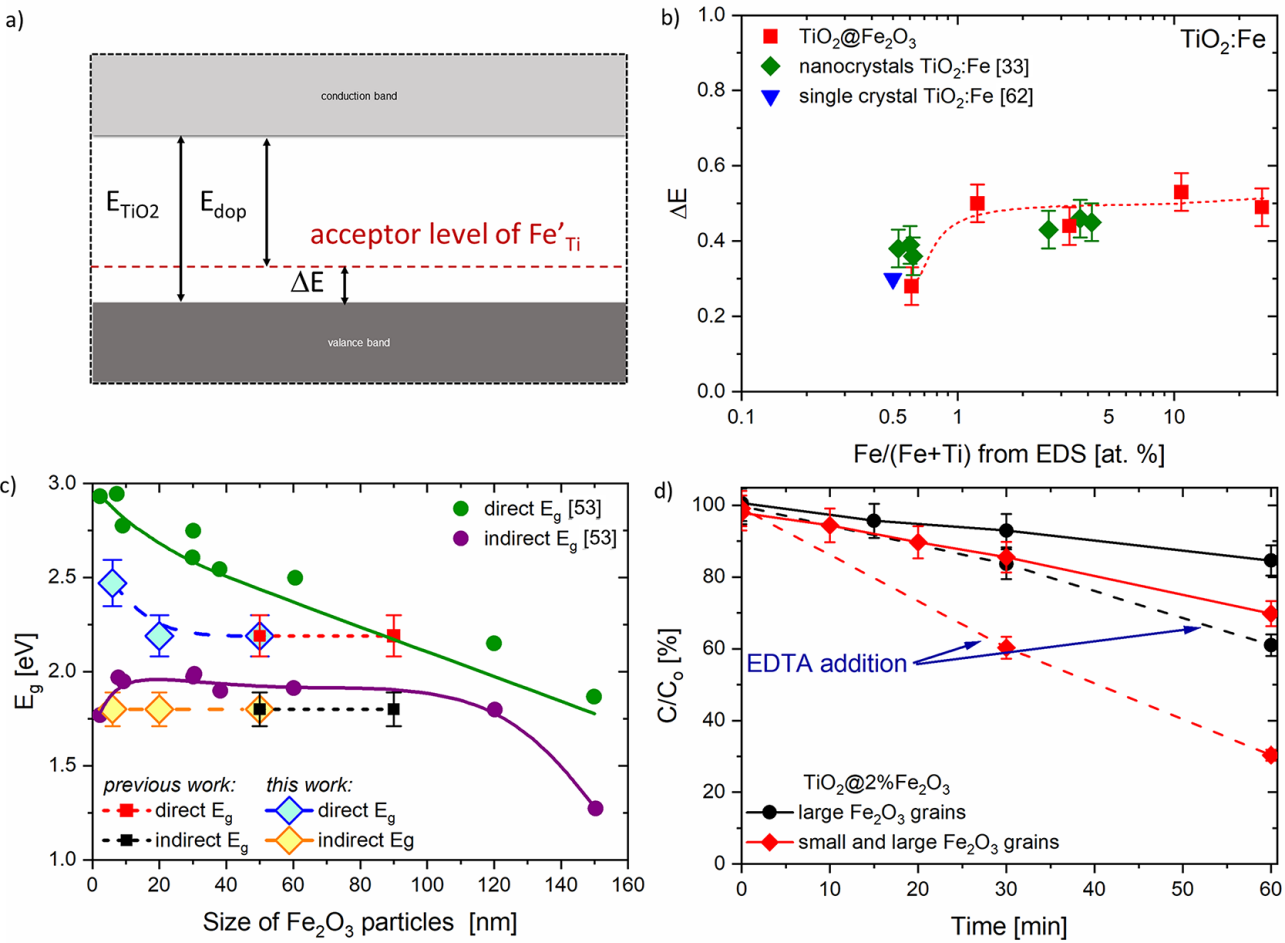
(a) Energy difference Δ*E* between the TiO_2_ nanocrystal band gap *E*_TiO_2__ and the acceptor doping level *E*_dop_ as a function of the Fe/(Fe + Ti) ratio obtained from EDX, our previous work,^33^ and single-crystal data from ref (59)*,* (b) acceptor level within the TiO_2_ band structure caused by Fe^3+^ doping, (c) dependence of the band gap energy E_g_ on the particle size of the hematite (ref (53)), and (d) TiO_2_@2%Fe_2_O_3_ nanostructure with different grain sizes of Fe_2_O_3_.

Not only optical results but also the analysis of the XPS studies support the possibility of substitution of some amount of Fe^3+^ into the titanium sublattice as Fe_Ti_^′^. The energy difference Δ*E* between the band gap of TiO_2_ nanocrystals (*E*_TiO_2__) and the acceptor doping level *E*_dop_ (Figure 10a) was calculated from the experimental data (Table S2) and is presented as a function of Fe/(Fe + Ti) in Figure 10b. The position of the iron Fe^3+^ level within the TiO_2_ band gap varies with the increasing Fe_2_O_3_ concentration. As can be seen, the Fe acceptor level is located in the range of 0.3–0.5 eV above the top of VB_TiO_2__ depending on the concentration of the dopants. It is also affected by the microstructural properties of titanium dioxide, that is, the form of material (nanopowders and nanocrystals) and the type of synthesis of TiO_2_@Fe_2_O_3_ heterojunctions.^33,53^ This effect has also been demonstrated for TiO_2_ modified with chromium Cr^3+^.^58^

The simultaneous occurrence of direct and indirect optical transitions has been demonstrated for α-Fe_2_O_3_,^52^ as discussed in Section 3.2 of this work and illustrated in Figure 10c. The well-pronounced size effect has been reported by Chernyshova et al.^53^ for direct optical transitions. The results of our studies regarding the absorption feature at 2.48 eV (3) indicate quite good correspondence with these observations (Figure 10c). This confirms that the size effect can be attributed to the presence of small 6–8 nm α-Fe_2_O_3_ nanograins, the evidence of which has been demonstrated by HRTEM.

**Figure 11:**
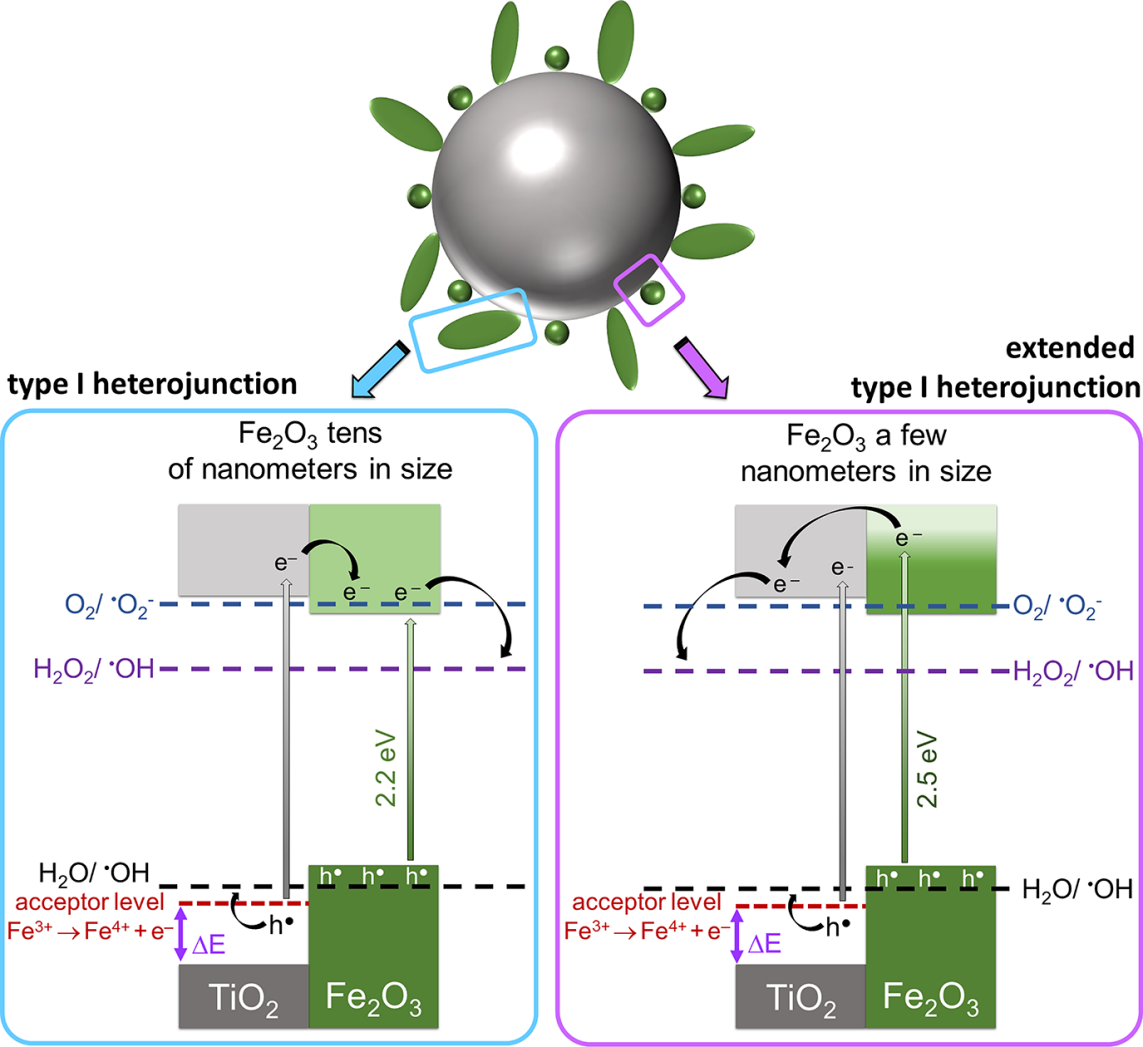
Proposed band diagram of the TiO_2_@Fe_2_O_3_ heterojunction and electron as well as hole transfer routes between electronic states.

The dye degradation (solid curves) presented in Figure 10d corresponds to the PS consisting of photocatalyst + RhB + H_2_O_2_. TiO_2_@2%Fe_2_O_3_ with different iron(III) oxide grain sizes were used as photocatalysts. It was observed that the RhB concentration for the photocatalyst with Fe_2_O_3_ grains of a large size decreased to 85% after 60 min, but the highest changes equal to 70% were observed for the TiO_2_@2%Fe_2_O_3_ heterojunction composed of both the large and small Fe_2_O_3_ grains after the same time. The explanation of this phenomenon is related to the different band structure at the interface caused by the different microstructure (see the explanation of Figure 11). Furthermore, when the hole scavenger was added (dashed lines, see Figure 10d), the decomposition accelerated due to the reduction of recombination at the TiO_2_@Fe_2_O_3_ interface (from 70 to 30% in the case of TiO_2_@2%Fe_2_O_3_).

Based on the experimental results, including optical, structural, and electronic properties, as well as HRTEM imaging, it was possible to propose the energy diagrams of the TiO_2_@Fe_2_O_3_ heterostructure presented in Figure 11 and the mechanism of RhB decomposition.

Covering of titanium dioxide with iron oxide is claimed to result in the formation of an additional acceptor level within the TiO_2_ band gap. Upon visible light irradiation, generation of electron–hole pairs can occur in TiO_2_ only when the acceptor level is activated (2.80 eV). Electrons excited from the Fe^3+^ level are transferred to the TiO_2_ CB following the reaction: Fe^3+^ → Fe^4+^ + e’. Centers Fe^4+^ can be treated as Fe^3+^ ions with photoholes h^•^ located on them. The holes can move in the TiO_2_ lattice by the hopping mechanism according to the reaction Fe^4+^ → Fe^3+^ + h^•^. The position of the Fe acceptor level is below the water oxidation potential H_2_O/^•^OH, while water oxidizes to hydroxyl radicals (eq 5) and then ^•^OH_(H_2_O)_ participates in RhB degradation (eq 2). However, it is a subsidiary reaction in this system.

In the previous work, hematite grains tens of nanometers in size, characterized by a direct band gap of 2.2 eV, formed at the TiO_2_ surface, and the type I heterojunction was created (Figure 11a). Furthermore, in this work, the obtained TiO_2_@Fe_2_O_3_ heterojunction possesses the same large Fe_2_O_3_ grains, but here, this interface has been carefully examined. In the type I heterojunction, the photoelectrons involved in the RhB decomposition originated from two sources. The first is the acceptor level formed in TiO_2_, from which the excited e^–^ are transferred to the CB of TiO_2_ and then to Fe_2_O_3_. The second are photoelectrons that form in the iron oxide (2.2 eV). Both participate in the reduction of hydrogen peroxide to OH^•^, and a small part of them reduced O_2_ to ^•^O_2_^–^. As mentioned in Section 3.3, hydroxyl radicals are the main active species in RhB degradation.

The extended type I heterojunction is created because of an additional optical transition at a photon energy of 2.5 eV that originated from Fe_2_O_3_ nanoparticles with a size of several nanometers. Photoelectrons from high energy levels in iron(III) oxide are transferred to lower energy states in the CB of titanium dioxide through the interface. Then, together with the electrons from TiO_2_, they reduce H_2_O_2_ to OH^•^, which is the main route of the decomposition of RhB.

## 5 Conclusions

The TiO_2_@Fe_2_O_3_ heterostructures composed of shape-controlled titanium dioxide nanocrystals covered with α-Fe_2_O_3_ nanoparticles have been successfully synthesized. The results of various characterization methods have shown that in addition to the presence of iron oxide nanoparticles on the surface of TiO_2_, the TiO_2_ lattice is substitutionally doped with Fe^3+^ ions, which is accompanied by the formation of oxygen vacancies. First, XPS studies of the O1peak have confirmed the existence of the component attributed to the oxygen vacancies V_O_ in the TiO_2_ lattice. Furthermore, the formation of a thin, doped TiO_2_:Fe layer has been found, manifested by the appearance of an additional acceptor level within the TiO_2_ band gap. In terms of the microstructure, SEM analysis revealed α-Fe_2_O_3_ nanoparticles of different shapes agglomerated in irregular grains up to 100 nm in size. However, deposition on the surface of TiO_2_ nanocrystals causes the crystallization of evenly distributed Fe_2_O_3_ nanoparticles with sizes several tens of nanometers (up to 50 nm from SEM) and a few nanometers (6–8 nm from HRTEM). The presence of Fe_2_O_3_ nanoparticles in TiO_2_@Fe_2_O_3_ heterostructures has also been evident in UV–vis studies, which have also shown an additional optical transition attributed to the size effect of α-Fe_2_O_3_. The photocatalytic performance of the TiO_2_ nanocrystals and heterostructures of TiO_2_@Fe_2_O_3_ toward RhB decomposition and the detailed mechanism of this reaction have been investigated using relevant scavengers to determine active species in the system. In-depth analysis has allowed the indication of ^•^OH hydroxyl radicals as the main active species responsible for the decomposition of RhB by TiO_2_ nanocrystals, Fe_2_O_3_ nanoparticles, and TiO_2_@Fe_2_O_3_ heterojunctions. On the basis of the experimental results and the relative band positions of the TiO_2_@Fe_2_O_3_ materials, the mechanism of RhB degradation was proposed. Under visible light, in addition to Fe_2_O_3_, only the Fe^3+^ acceptor level within the TiO_2_ band gap is active, and electron–hole pairs are created. Electrons excited from the Fe^3+^ acceptor level are transferred to the TiO_2_ CB. Furthermore, the high energy levels located in the Fe_2_O_3_ CB associated with the optical transition are responsible for the electron transfer from CB_Fe2O3_ to CB_TiO2_. Therefore, all electrons in the TiO_2_ CB participate in the formation of OH radicals in the reaction with H_2_O_2_, which is considered the most probable route of RhB decomposition. The proposed band diagram of the TiO_2_@Fe_2_O_3_ heterojunction supports the hypothesis of an extended type I band configuration.

## Supporting Information Available

EDX analysis of chemical composition; XRD pattern of the TiO_2_ nanocrystal; TEM images of the TiO_2_@Fe_2_O_3_ heterostructure; EDX mapping and HRTEM images with FFT and IFFT analysis of the TiO_2_@Fe_2_O_3_ heterostructure; XPS analysis of the TiO_2_@Fe_2_O_3_ heterostructure; analysis of spectral dependence of total reflectance and optical transition energies of TiO_2_ and the TiO_2_@Fe_2_O_3_ heterostructure; analysis of photocatalytic decomposition of RhB dye under visible radiation, photocatalytic activity with and without the addition of H_2_O_2_ to the PS; dye degradation in the presence of scavenger of holes; and recycled photocatalytic decomposition process of RhB in the presence of TiO_2_@Fe_2_O_3_ and H_2_O_2_ (PDF)
