# Supplementary material for: Scavenger-Supported Photocatalytic Evidence of an Extended Type I Electronic Structure of the TiO2@Fe2O3 Interface
Source: ACS Appl Mater Interfaces. 2022 Aug 15;14(33):38255–69. doi: 10.1021/acsami.2c06404 (PMC9412959; doi:10.1021/acsami.2c06404)
Supplement: Supplementary file 1 — am2c06404_si_001.pdf [file am2c06404_si_001.pdf]

Supporting Information

For

Scavenger-supported photocatalytic evidence of an extended type I electronic structure of TiO_2@Fe_2O_3 interface

Anita Trenczek-Zajac[*1], *Milena Synowiec*[*1], *Katarzyna Zakrzewska*[2], *Karolina Zazakowny*[1], *Kazimierz Kowalski*[3], *Andrzej Dziedzic*[4] *and Marta Radecka*[1]

[1]Faculty of Materials Science and Ceramics, AGH University of Science and Technology, Krakow, Poland,

[2]Faculty of Computer Science, Electronics and Telecommunications, AGH University of Science and Technology, Krakow, Poland

[3]Faculty of Metals Engineering and Industrial Computer Science, AGH University of Science and Technology, Krakow, Poland

[4]Institute of Physics, College of Natural Sciences, University of Rzeszow, Rzeszow, Poland

***Corresponding author:**

milsyn@agh.edu.pl, anita.trenczek-zajac@agh.edu.pl

KEYWORDS

TiO_2, Fe_2O_3, heterostructures, band diagram, interface, electron transfer, photocatalysis

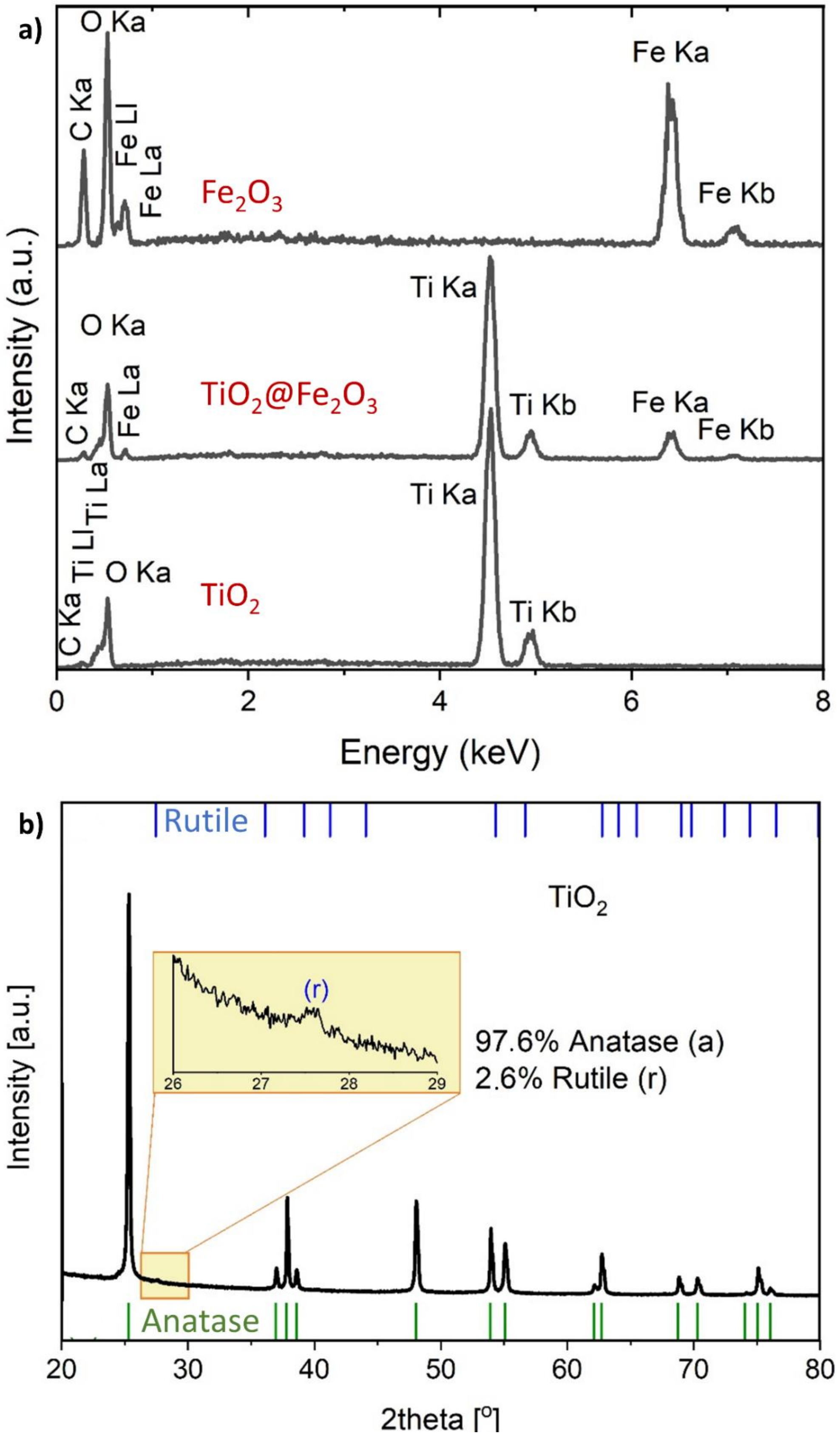

Figure S1. a) EDX spectrum of selected nanomaterials, b) X-ray diffraction pattern of TiO_2 nanocrystals with traces of rutile.

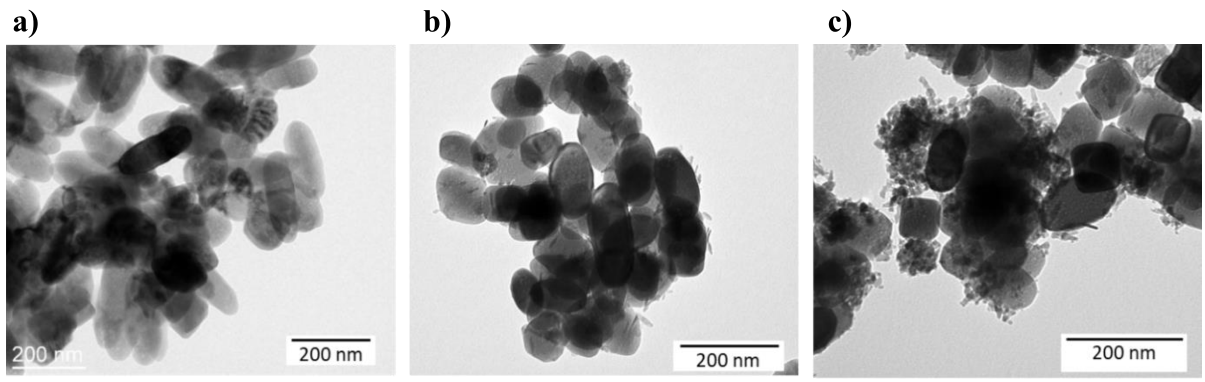

Figure S2. TEM images of a) TiO_2@2%Fe_2O_3, b) TiO_2@10%Fe_2O_3, c) TiO_2@20%Fe_2O_3.

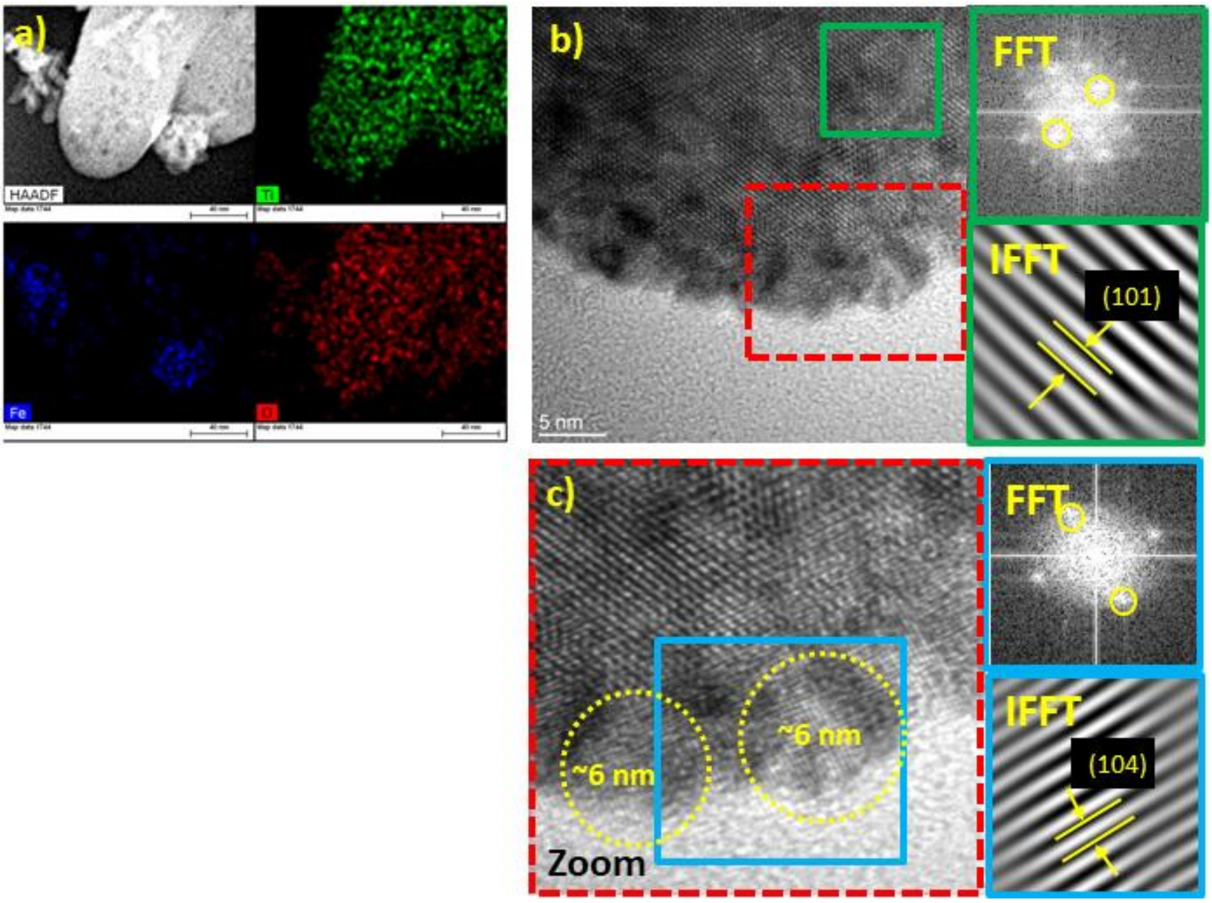

Figure S3. TiO_2@20%Fe_2O_3 sample a) EDX mapping images; b) HRTEM images with Fast Fourier Transform and IFFT analysis indicate the existence of (101) plane of anatase TiO_2; c) FFT of the blue rectangle from the zoomed area shows hematite nanoparticles of 6 nm size with the (104) plane of α-Fe_2O_3.

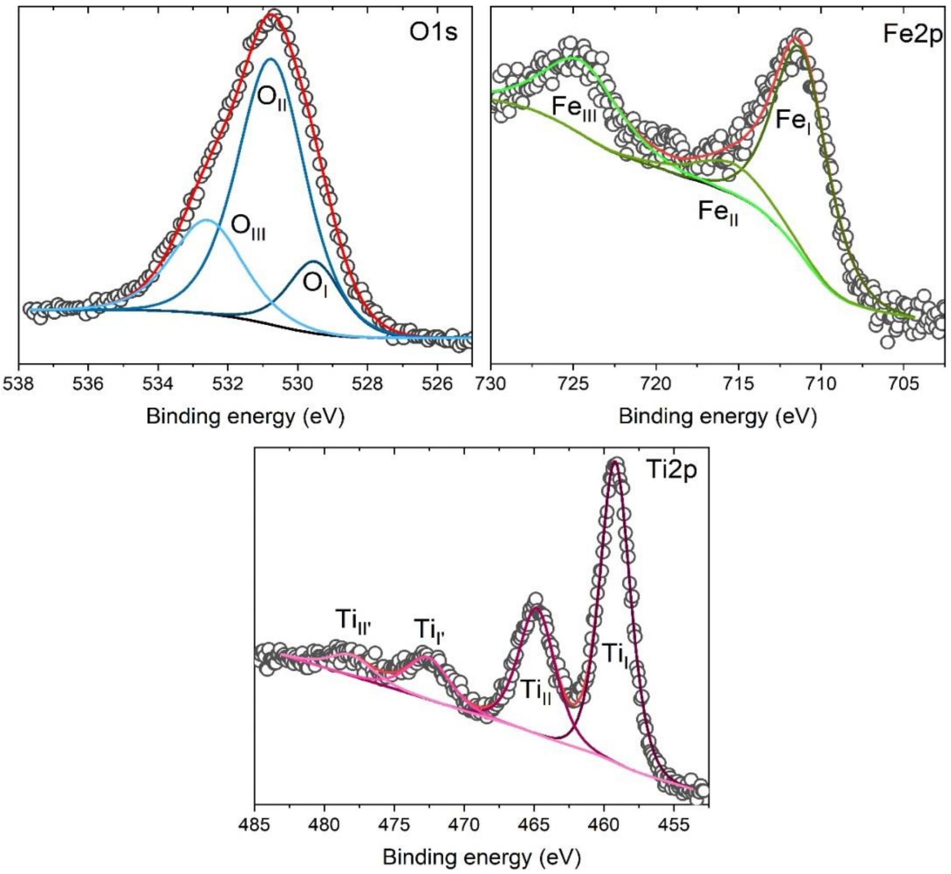

Figure S4. Deconvolution of the XPS spectra of O1s, Ti2p and Fe2p of TiO_2 nanocrystals heavily modified with Fe_2O_3 (TiO_2@20%Fe_2O_3).

Table S1. Characteristic binding energies of Ti2p, O1s, and Fe2p determined from XPS analysis for TiO_2 heavily modified with Fe_2O_3 (TiO_2@20%Fe_2O_3).

Symbol	Binding energy (eV)	Type of bonding	Ref.
	**Ti2p**		
Ti_I	459.2(3)	Ti2$p_{3/2}$, O-Ti-O	[1]
Ti_{II}	464.9(3)	Ti2$p_{1/2}$, O-Ti-O	[1]
$Ti_{I'}$	472.7(3)	satellite of Ti2$p_{3/2}$, O-Ti-O	[1]
$Ti_{II'}$	478.3(3)	satellite of Ti2$p_{1/2}$, O-Ti-O	[1]
	**O1s**		
O_I	529.5(4)	Ti-O-Ti	[2]
O_{II}	530.8(4)	oxygen vacancies or defects	[2]
O_{III}	532.8(4)	chemisorbed species e.g. OH^-, H_2O, O^{2-}	[2]
	**Fe2p**		
Fe_I	711.5(5)	Fe2$p_{3/2}$, Fe^{3+} in Fe_2O_3	[3–5]
Fe_{II}	715.6(5)	satellite of Fe2$p_{3/2}$, Fe^{3+} in Fe_2O_3	[3–5]
Fe_{III}	724.8(5)	Fe2$p_{1/2}$, Fe^{3+} in Fe_2O_3	[3–5]

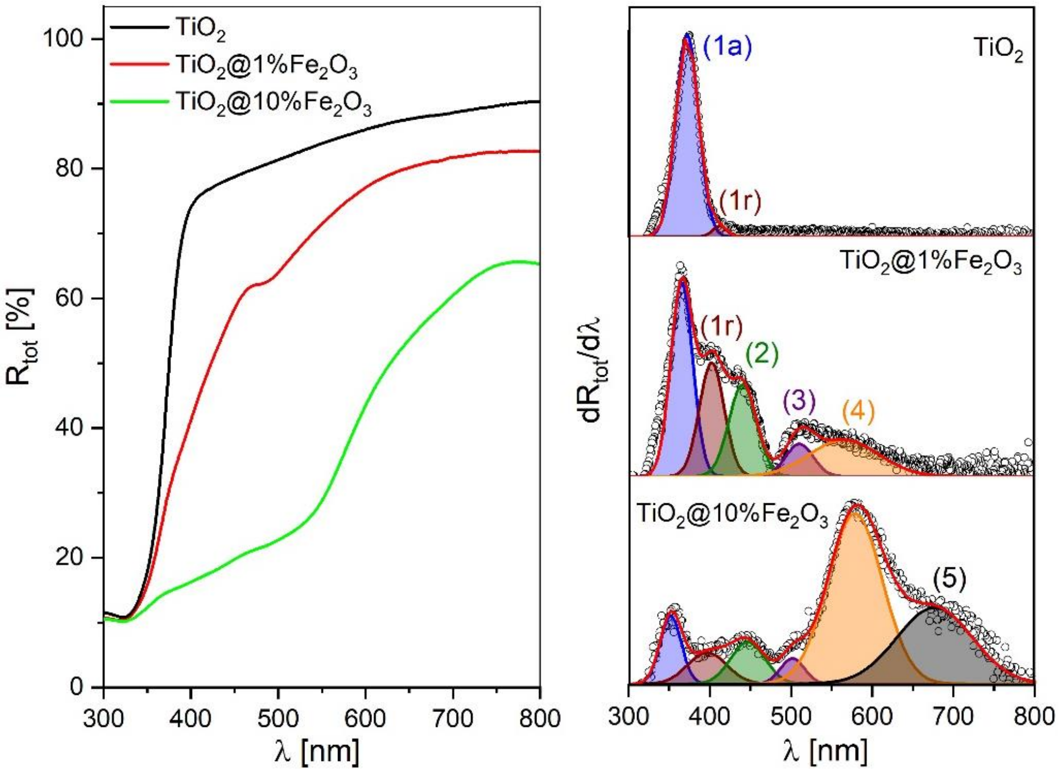

Figure S5. Spectral dependence of total reflectance (left-hand side) and first derivative spectra $dR_{tot}/d\lambda$ (right-hand side) for TiO_2 NCs before and after modification with Fe_2O_3.

Table S2. Energies of the optical transitions for TiO_2 and TiO_2@Fe_2O_3 nanomaterials obtained from the first derivative plot. Transition energies were determined with an uncertainty of 0.02 eV.

**Sample**	**(1a) (eV)**	**1r (eV)**	**(2) (eV)**	**(3) (eV)**	**(4) (eV)**	**(5) (eV)**
(TiO_2)*	3.32	-	-	-	-	-
TiO_2	3.34	3.04				
(TiO_2@0.2%Fe_2O_3)*	3.36	-	3.02	-	-	-
TiO_2@1%Fe_2O_3	3.40	3.08	2.82	2.43	2.19	--
(TiO_2@2%Fe_2O_3)*	3.43	-	2.92	2.48	2.17	1.84
TiO_2@10%Fe_2O_3	3.54	3.14	2.79	2.48	2.15	1.84
(TiO_2@20%Fe_2O_3)*	3.52	-	2.81	2.48	2.13	1.81
Fe_2O_3	-	-	-	-	2.11	1.78

* In this case, TiO_2 nanocrystals appears only as anatase phase.

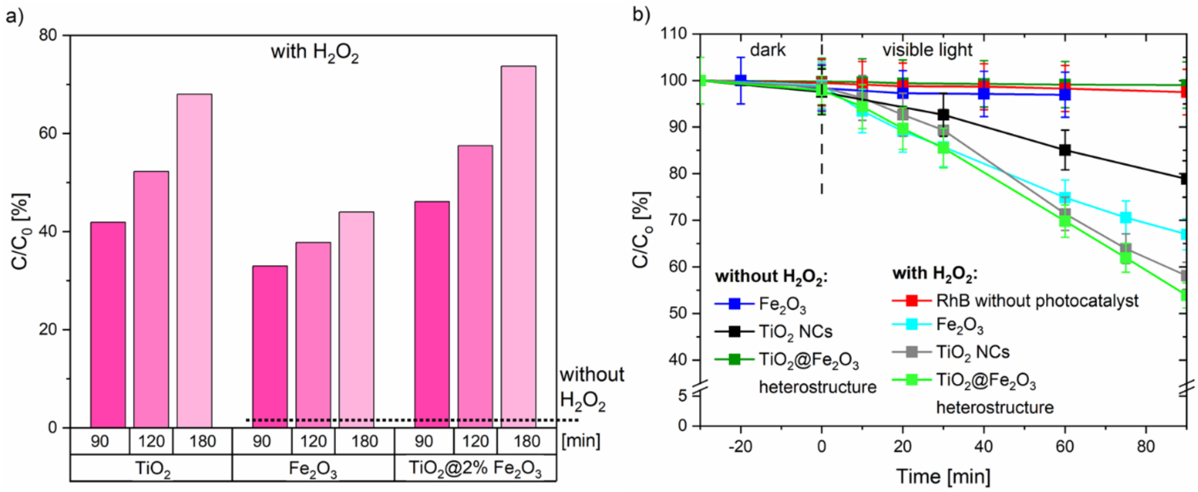

Figure S6. (a) Photocatalytic decomposition of RhB dye under visible radiation for pure oxides vs. TiO_2@2%Fe_2O_3 heterojunction, (b) comparison of photocatalytic activity with and without addition of H_2O_2 to the photocatalytic system.

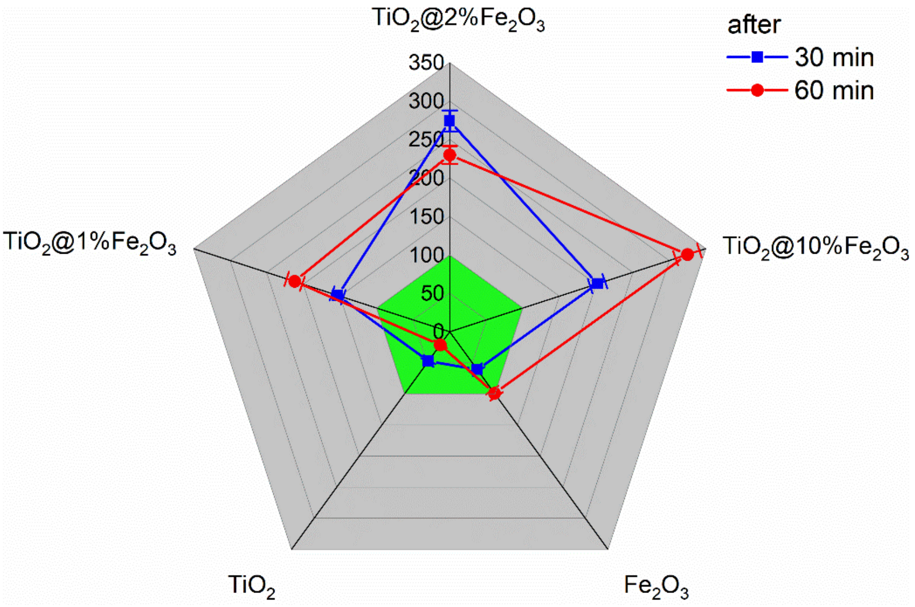

Figure S7. Normalized dye degradation after 30 and 60 min, in the presence of scavenger of holes.

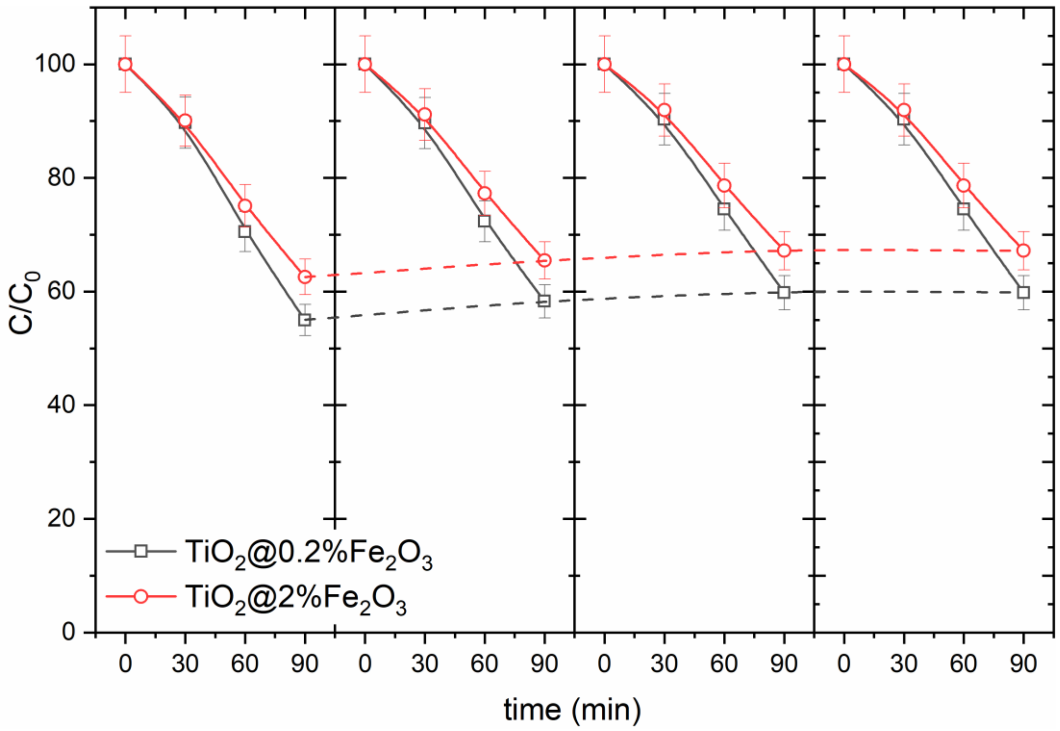

Figure S8. Recycled photocatalytic process of Rhodamine B in the presents of $TiO_2@Fe_2O_3$ and H_2O_2.

References

1. Saha, N.C.; Tompkins, H.G. Titanium Nitride Oxidation Chemistry: An X-ray Photoelectron Spectroscopy Study. *J. Appl. Phys.* **1992**, *72*, 3072–3079, doi:10.1063/1.351465.
2. Ghobadi, A.; Ulusoy, T.G.; Garifullin, R.; Guler, M.O.; Okyay, A.K. A Heterojunction Design of Single Layer Hole Tunneling ZnO Passivation Wrapping around TiO2 Nanowires for Superior Photocatalytic Performance. *Sci. Rep.* **2016**, *6*, 30587, doi:10.1038/srep30587.
3. Bajnóczi, É.G.; Balázs, N.; Mogyorósi, K.; Srankó, D.F.; Pap, Z.; Ambrus, Z.; Canton, S.E.; Norén, K.; Kuzmann, E.; Vértes, A.; et al. The Influence of the Local Structure of Fe(III) on the Photocatalytic Activity of Doped TiO2 Photocatalysts—An EXAFS, XPS and Mössbauer Spectroscopic Study. *Appl. Catal. B Environ.* **2011**, *103*, 232–239, doi:10.1016/j.apcatb.2011.01.033.
4. Yamashita, T.; Hayes, P. Analysis of XPS Spectra of Fe2+ and Fe3+ Ions in Oxide Materials. *Appl. Surf. Sci.* **2008**, *254*, 2441–2449, doi:10.1016/j.apsusc.2007.09.063.
5. Moradi, V.; Jun, M.B.G.; Blackburn, A.; Herring, R.A. Significant Improvement in Visible Light Photocatalytic Activity of Fe Doped TiO2 Using an Acid Treatment Process. *Appl. Surf. Sci.* **2018**, *427*, 791–799, doi:10.1016/j.apsusc.2017.09.017.
